# Mycorrhizal inoculation modulates metabolism and gene networks to enhance salinity tolerance in quinoa

**DOI:** 10.3389/fpls.2026.1831758

**Published:** 2026-05-20

**Authors:** Soumaya Zaidi, Abdelilah Meddich, Marouane Baslam, Anja Hartmann, Nicolaus von Wirén, Mohammad-Reza Hajirezaei

**Affiliations:** 1 Department of Physiology and Cell Biology, Leibniz-Institute of Plant Genetics and Crop Plant Research (IPK), Gatersleben, Germany; 2 Department of Physiology and Cell Biology, Center of Agrobiotechnology and Bioengineering, Research Unit Labeled National Center for Scientific and Technical Research (CNRST) (Centre AgroBiotech-URL-CNRST-05), “Physiology of Abiotic Stresses” Team, Cadi Ayyad University, Gatersleben, Marrakesh, Morocco; 3 African Sustainable Agriculture Research Institute (ASARI), Mohammed VI Polytechnic University (UM6P), Laâyoune, Morocco; 4 GROWSMART Inc., Seoul, Republic of Korea

**Keywords:** arbuscular mycorrhizal fungi (AMF), carbon metabolism, metabolic alterations, molecular responses, osmotic adjustment, quinoa (*Chenopodium quinoa*), salinity stress

## Abstract

Arbuscular mycorrhizal fungi (AMF) are increasingly recognized for their role in enhancing tolerance to salinity stress. Here, we present an integrative analysis combining metabolic, ionic, and transcriptomic profiling to investigate early vegetative-stage responses of quinoa (*Chenopodium quinoa* Willd.) to salinity and AMF inoculation, an approach that remains underexplored. Plants were exposed to different salinity levels with or without AMF inoculation, and growth, chlorophyll concentration, ion accumulation, metabolite profiles, and gene expression patterns were analyzed. AMF-inoculated plants displayed slightly enhanced growth, reduced Cl^-^ accumulation, and significantly higher chlorophyll (Chl) levels under high salinity. Metabolomic analysis revealed a shift in carbon flux toward Chl biosynthesis, characterized by decreased glucose levels and sustained accumulation of key intermediates and Chl precursors, including 3-phosphoglycerate (3PGA), phosphoenolpyruvate (PEP), 2-oxoglutarate, malate, oxalic acid, and glutamate. These changes were accompanied by increased levels of amino acids (alanine, isoleucine, proline) and phenolic compounds (ferulic acid, benzoic acid, and p-coumaric acid), suggesting improved osmotic adjustment and stress tolerance. Transcriptomic analysis revealed upregulation of genes involved in Chl biosynthesis (*GLK1*, *PORA*) and enrichment of pathways related to carbon metabolism, photosynthesis, starch and sucrose metabolism, and the tricarboxylic acid cycle in AMF-inoculated plants. In addition, stress-responsive genes (e.g., *CMO*, aspartic proteinase inhibitors, CBSCBS2) were upregulated, while other stress-signaling genes (*C2H2-ZFP*, *PYL4*) were repressed. Weighted gene co-expression network analysis further identified gene modules correlated with metabolites and traits related to Chl biosynthesis, carbon metabolism, and stress-responsive pathways. Overall, our findings indicate that AMF inoculation is associated with coordinated metabolic and transcriptional adjustments that contributed to enhanced salinity tolerance in quinoa.

## 1 Introduction

Soil salinity is an increasing threat to global agriculture, affecting over 20% of irrigated lands and undermining food production in arid and semi-arid regions (FAO, 2021). As salt accumulates, it disrupts water uptake, ion balance, and carbon assimilation, limiting plant growth and yield. At the plant level, salinity causes physiological, biochemical, and molecular changes (Arif et al., 2020; Roșca et al., 2023). Salt ions, such as sodium (Na^+^) and chloride (Cl^-^), bind water molecules, causing osmotic stress. Their excessive accumulation disrupts ion uptake, leading to nutrient disorders and leaf necrosis (Bezerra-Neto et al., 2022). Additionally, salinity induces oxidative stress by overproducing reactive oxygen species (ROS), damaging enzymes, membranes, and cellular functions (Bose et al., 2014). These stresses collectively impair plant growth and productivity. To counteract salinity, plants utilize mechanisms such as the accumulation of osmolytes and osmoprotectants, including amino acids, sugars, organic acids, and phenolics, which help maintain osmotic balance, hydration, and photosynthesis. Plants also export Na^+^, compartmentalize excess ions, and activate antioxidant systems to reduce oxidative damage (Minh et al., 2016; Arif et al., 2020). At the molecular level, salinity stress increases the expression of various genes and transcription factors to safeguard ion homeostasis and enhance tolerance to salinity (Mansour and Hassan, 2022).

Beyond intrinsic stress tolerance mechanisms, plants can also benefit from soil microbes, which enhance resilience by mitigating environmental stressors. Arbuscular mycorrhizal fungi (AMF), an essential part of the soil microbiota, improve water and nutrient uptake, reducing the need for synthetic fertilizers (Bhupenchandra et al., 2024). AMF form symbiotic relationships with most terrestrial plants by developing arbuscules within root cortical cells. These fungi rely on the delivery of carbohydrates from their host plants to support their growth, while enhancing the plant’s ability to acquire water and essential nutrient elements. Under salinity stress, AMF symbiosis improves not only nutrient and water uptake but also strengthens the plant’s stress response (Evelin et al., 2019). Studies have shown that AMF enhances photosynthetic capacity and induces metabolic changes in the roots and shoots of plants such as wheat and date palm (Ait-El-Mokhtar et al., 2020; Huang et al., 2023). Furthermore, AMF symbiosis has been shown to upregulate several stress-responsive genes, thereby further enhancing plant adaptability to saline environments (Evelin et al., 2019).

While AMF-mediated growth promotion and physiological benefits have been extensively studied in salt-sensitive plants, research on salt-tolerant species remains limited, particularly at the molecular, transcriptomic, and metabolomic levels. Nevertheless, a few studies have demonstrated beneficial AMF effects in salt-tolerant crops and halophytes, including barley, Suaeda salsa, and Asteriscus maritimus (Estrada et al., 2013; Diao et al., 2021; Slimani et al., 2024). These findings suggest that AMF can support salt-tolerant species, but the underlying mechanisms may differ from those observed in glycophytes. As noted by Pan et al. (2020), salt-tolerant species often possess distinct physiological traits, such as ion-based osmotic adjustment and efficient nutrient uptake, which could shape their interactions with AMF in unique ways.

Among salt-tolerant crops, quinoa (*Chenopodium quinoa* Willd.) stands out for its exceptional adaptability to saline environments and growing agricultural relevance (Gordillo-Bastidas et al., 2016). Yet, despite its physiological tolerance, the role of AMF in modulating quinoa’s metabolic, ionic, and molecular responses to salinity stress remains largely unexplored. Investigating this interaction is therefore critical to deepen our understanding of how AMF contribute to stress adaptation in inherently salt-tolerant crops.

Here, we hypothesize that AMF enhances quinoa’s salinity tolerance at the early growth stage by altering carbon metabolism, osmoprotection, and stress-responsive gene networks. Using a multi-omics approach, we explore how AMF modulates metabolic pathways and gene expression to maintain chlorophyll (Chl) biosynthesis and ion homeostasis under salinity. This approach provides new insights into quinoa-AMF interactions.

## 2 Materials and methods

### 2.1 Plant material and mycorrhizal inoculum preparation

Quinoa seeds (cv. Titicaca) were surface sterilized with 96% ethanol for 30 s, followed by 5% sodium hypochlorite for 10 min, and rinsed thoroughly with distilled water. The sterilized seeds were germinated for 2 weeks in a sterile substrate (Substrate 1, Klasmann-Deilmann GmbH, Germany) under controlled conditions (day/night temperatures of 14/12 °C, relative humidity of 85%, and light intensity of 250–300 µE). The seedlings were then vernalized for three weeks under controlled conditions (5 °C, 10 h light/14 h dark photoperiod, 80% relative humidity) before treatment application. Seed germination and vernalization were conducted over a total period of approx. five weeks, from 25 November 2022 to 1 January 2023.

Mycorrhizal spores were isolated from a palm grove in the Tafilalet region of southeastern Morocco, where 15 species were identified: *Acaulospora delicata*, *A. laevis*, *Acaulospora* sp., *Claroideoglomus claroideum*, *Glomus aggregatum*, *G. claroides*, *G. clarum*, *G. deserticola*, *G. heterosporum*, *G. macrocarpum*, *G. microcarpum*, *Glomus* sp., *G. versiforme*, *Rhizophagus intraradices*, and *Pacispora boliviana* (Benaffari et al., 2022). The AMF inoculum was produced by propagating a mixture of spores from these isolates in *Zea mays* L. (cv. Anasazi Sweetcorn) roots for three months in sandy soil. The resulting soil, containing infected root fragments, mycelia, and spores (386 spores per 10 g of soil and approx. 80% colonization), was collected and used as AMF inoculum in this study.

### 2.2 Experimental setup and treatments

Following five weeks of germination and vernalization, the seedlings were transplanted into 1.5-kg pots on 2 January 2023, at which point AMF inoculum was applied. Four treatments were established: AMF-inoculated plants received either a lower inoculum level (20 g, corresponding to 772 spores per pot; AMF20) or a higher inoculum level (50 g, corresponding to 1930 spores per pot; AMF50), while non-inoculated plants (C20 and C50) received the corresponding amounts of sterilized inoculum. The selected inoculum levels were based on a previous experiment, in which a lower spore density (10 g, corresponding to 386 spores per pot) had no significant effect on quinoa growth under salinity (**Supplementary Table 1**), necessitating the use of higher inoculum levels to evaluate potential dose-dependent effects. In that experiment, conditions were comparable to those in the present study, with plants subjected to 0, 200, and 400 mM NaCl for 20 days, and AMF inoculum was applied 1 week before the onset of salinity stress. The use of sterilized inoculum in control treatments ensured that any observed plant responses were attributable to AMF biological activity rather than to the physical or chemical properties of the inoculum. The substrate used for this experiment consisted of a nutrient-free substrate (Einheitserde Typ 0, H. Nitsch & Sohn GmbH, Germany) and substrate 1 mixed in a 1:6 ratio, resulting in a total phosphorus (P) content of 50 mg.kg^-1^. All substrates were sterilized by autoclaving twice at 120 °C for 20 minutes, with a 4-day interval between cycles.

After transplantation, plants were maintained in a greenhouse under controlled conditions (16 h light/20 °C, 8 h dark/16 °C) for 16 days (2–17 January 2023) prior to stress application, to allow AMF colonization and establishment of the symbiotic interaction. During this period, plants were watered daily with 200 mL of distilled water and fertilized once per week using WUXAL Top N fertilizer (Wilhelms GmbH, Germany). Pots were arranged in a fully randomized design, with positions changed weekly. Each treatment consisted of six replicates, resulting in a total of 72 pots.

Salinity stress was initiated on 18 January 2023 and maintained for 11 days (until 29 January 2023) by daily watering with 200 mL of sodium chloride (NaCl) solution at three levels: no salinity (0 mM), moderate (100 mM), and high (200 mM). A concentration of 100 mM NaCl is generally tolerated by quinoa with adaptive responses, whereas 200 mM NaCl represents a threshold at which marked growth inhibition occurs, according to previous studies (Cai and Gao, 2020; Rodríguez-Hernández et al., 2021). The 11-day stress duration was selected to capture early vegetative-stage responses to salinity, enabling the detection of morphological, metabolic, and transcriptional changes before the onset of severe damage. This is supported by a previous study showing that salinity stress in quinoa can have significant effects within a short time frame, with measurable growth and physiological alterations reported after 7 days at 200 mM NaCl (Rodríguez-Hernández et al., 2021). In addition, early AMF-mediated metabolic and transcriptomic responses under salinity have been documented within similar timeframes (Qin et al., 2021).

Plants were harvested on 29 January 2023, at 62 days after sowing. Shoot dry biomass and plant height were measured. Leaf discs (0.7 cm diameter) and fully expanded leaf material were collected, immediately frozen in liquid nitrogen, and stored at −80 °C for subsequent metabolomic and RNA-seq analyses. For elemental analysis, frozen leaf material and thoroughly rinsed root samples were dried at 65 °C for four days. A subset of three biological replicates of root material from each plant was stored at −4 °C for mycorrhizal quantification.

### 2.3 Estimation of mycorrhizal colonization

AMF colonization frequency and intensity were assessed following established methods for root clearing and staining (Vierheilig et al., 1998) and quantified according to Trouvelot et al. (1986). Roots were cleared in 10% potassium hydroxide (KOH) at 60 °C for 30 min, acidified in 2 N hydrogen chloride for 30 s, and stained with a 5% ink-acid solution at 60 °C for 40 min. The roots were then destained in lactic acid for two weeks. After cutting the roots into 1 cm fragments, 60 fragments were observed individually under a light microscope. Colonization frequency (CF) and intensity (CI) were calculated according to Trouvelot et al. (1986):

CF (%) = (Number of colonized fragments/Total fragments observed) × 100.

CI (%) = (95n_5_ + 70n_4_ + 30n_3_; + 5n_2_ + n_1_)/Total number of observed root fragments.

Root fragment ratings (0–5) were assigned according to Trouvelot et al. (1986) as follows: 0 = no colonization, 1 = trace colonization, 2 = less than 10% colonization, 3 = 11-50% colonization, 4 = 51-90% colonization, 5 = 91% or more colonization.

### 2.4 Determination of total Chl concentration

Chl pigments were extracted by incubating 0.7 cm leaf discs in 1 mL of 80% acetone for 60 min. Chl a, Chl b, and total Chl (Chl a+b) concentrations were measured using a spectrophotometer at 647 nm and 664 nm, with quantification based on Roca et al. (2016). Results were expressed per unit area (mg cm^-2^).

Chl a = 1000× ((12.7 × A_664_) − (2.55 × A_647_)).

Chl b = 1000 × ((20.31 × A_647_) − (4.91 × A_664_)).

Total Chl = 1000 × ((17.76 × A_647_) + (7.34 × A_664_)).

### 2.5 Determination of elemental composition

For elemental analysis, leaf and root samples were dried at 65 °C for 4 days, homogenized, and ground into a fine powder. For nitrogen (N) analysis, 1.5 mg of the dried powder was weighed into tin capsules and analyzed using a EuroEA3000 elemental analyzer (EuroVector SpA, Italy) with Callidus 5.1 software (Analytik Jena GmbH, Germany), following standard procedures for elemental analysis (Bremner, 1996).

Macro- and micronutrient concentrations were determined following established acid digestion and ICP-OES procedures (Jones and Case, 1990). Briefly, 10–15 mg of dried material was digested in 1 mL of concentrated nitric acid (67–69%) in PTFE tubes, followed by pressurization in a microwave reactor (traCLAVE IV, MLS GmbH). The digested material was diluted to 15 mL with ultrapure water, and elemental analysis was performed using ICP-OES (iCAP 7400 duo OES spectrometer, Thermo Fisher Scientific, Germany).

### 2.6 Extraction and quantification of carbohydrates and amino acids

To analyze soluble sugars, starch, and amino acids in leaf tissue, a modified protocol based on Tula et al. (2020) was used. About 50 mg of frozen leaf powder was homogenized in 0.7 mL of 80% ethanol and incubated at 80 °C with shaking at 800 g for 1 h, followed by centrifugation at 15, 000 g for 15 min at 4 °C. The supernatant was evaporated using a Speed-Vac system (Christ RVC2-33IR, Germany) at 40 °C for 2 h. The residue was dissolved in 0.3 mL ultrapure water, centrifuged, and analyzed for carbohydrates and amino acids. Glucose, fructose, and sucrose levels were quantified using a coupled photometric assay that monitored NADH oxidation at 340 nm (Tula et al., 2020).

Amino acid analysis was conducted using ultra-high-performance liquid chromatography (UPLC) on an Acquity H-Class system (Waters, Germany) with a fluorescence detector. Leaf extracts were derivatized using 6-aminoquinolyl-N-hydroxysuccinimidyl carbamate (AQC) (Bioanalytics, Gatersleben, Germany). For derivatization, 10 μL of leaf extract was mixed with 10 μL of AQC solution and 80 μL of 0.2 M boric acid (pH 8.8), then incubated at 55 °C for 10 min. Amino acids were separated on a Luna Omega C18 column (100×2.1 mm, 1.6 μm, Phenomenex) with a flow rate of 0.6 mL·min^-1^ over a 6-minute run at 40 °C according to the manufacturer’s instructions (Bioanalytics, Gatersleben, Germany). Detection occurred at 266 nm (excitation) and 473 nm (emission). Quantification was based on calibration curves of 19 amino acids (1–100 µM) with R² values >0.98. Data analysis was performed using Empower 3 software (Waters GmbH, Germany).

### 2.7 Extraction and quantification of primary metabolites

Primary metabolites, including central carbon metabolism intermediates and organic acids, were extracted following Ghaffari et al. (2016) with modifications. Approximately 100 mg of frozen leaf powder was homogenized in 1 mL of LC-MS grade methanol/chloroform (1:1) solution at 4 °C for 20 min (Th. Geyer GmbH & Co. KG, Renningen, Germany). After adding 0.3 mL ultrapure water, samples were centrifuged at 15, 000 g for 15 min at 4 °C. The supernatant was transferred to fresh tubes and dried using a Speed-Vac concentrator at 40 °C for 2–3 h. The residue was resuspended in 0.25 mL ultrapure water and shaken for 15 min at 4 °C for metabolite quantification. Metabolite separation and detection were carried out using an ion chromatography-mass spectrometry (IC-MS/MS) system, as described by Ghaffari et al. (2016). The system included a conductivity detector (Dionex Thermo Fisher Scientific, Germany) coupled with an Agilent 6495 Triple Quadrupole LC/MS System (Agilent Technologies, Germany). Anionic compounds were separated using a Dionex IonPac AS11-HC analytical column (250×2 mm), connected to a Dionex IonPac AG11-HC guard column (50×2 mm), and an ATC-1 anion trap column. Gradient elution was performed using ultrapure water (buffer A) and concentrated KOH (buffer B) via an EG-SP eluent generator (Dionex). The column was equilibrated at 0.32 mL·min^-1^ and heated to 35 °C. Electrospray ionization tandem mass spectrometry (ESI-MS/MS) was conducted in negative ion mode, with nitrogen gas at 12 L·min^-1^, a heating temperature of 250 °C, and a nebulizer pressure of 35 psi. The capillary voltage was set at 3 kV, with a dwell time of 20 ms. Collision energies were adjusted between 1 and 80 eV depending on the mass-to-charge ratios. Multiple reaction monitoring (MRM) was used for accurate compound identification and quantification. All analyzed compounds were quantified using calibration curves based on standards with concentrations ranging from 25 µM to 500 µM. Data acquisition was performed using Chromeleon (version 7.3, Dionex) and Agilent MassHunter LC/MS Acquisition software (B.07.01, Agilent Technologies), with quantification via MassHunter Quantitative Analysis software (B10.1, Agilent Technologies).

### 2.8 Extraction and quantification of phenolic compounds

Phenolic compounds, including p-coumaric acid, ferulic acid, and benzoic acid, were extracted using a modified method from Irakli et al. (2021). About 100 mg of frozen leaf powder was mixed with 1 mL of 80% methanol and sonicated at 30 °C for 1 h. The extract was then centrifuged at 15, 000 g for 10 min at 4 °C. The supernatant was transferred to new tubes, evaporated using a Speed-Vac concentrator at 40 °C for 2 h, and the residue resuspended in 0.1 mL of a 1:1 methanol-water solution for Ultrapressure Liquid Chromatography-Mass Spectrometry (UPLC-MS/MS) analysis.

UPLC-MS/MS analyses were performed using an Agilent 1290 Infinity II UHPLC system coupled with an Agilent 6495 Triple Quadrupole LC/MS System (Agilent Technologies, Germany). Chromatographic separation was achieved with an Eclipse Plus C18 RRHD column (50 × 2.1 mm, 1.8 μm, Agilent Technologies, USA) at a flow rate of 0.25 mL·min^-1^ and 40 °C. Two microliters of each sample were injected and eluted using solvent A (water) and solvent B (acetonitrile), both containing 0.1% formic acid (v/v). ESI-MS/MS was performed in negative ionization mode with nitrogen as the drying and nebulizing gas, set at 12 L·min^-1^, 250 °C, and a nebulizer pressure of 30 psi. The capillary voltage was maintained at 2 kV, and the dwell time was set to 20 ms. Collision energies ranged from 1 to 45 eV, optimized for each compound using MassHunter Optimizer software in MS2 SIM mode. MRM was used to target parent and daughter ions of the metabolites of interest. A total of 9 phenolic compounds were quantified (**Supplementary Table 2**), with calibration curves prepared using standards from 0.1 to 100 µM. Data acquisition and analysis were performed using Agilent MassHunter LC/MS Acquisition software (B.07.01) and MassHunter Quantitative Analysis (B10.1) software.

### 2.9 RNA isolation and analysis

Total RNA was isolated from leaf tissue using the innuPREP Plant RNA Kit (Analytik Jena, Germany) according to the manufacturer’s instructions. Three biological replicates were analyzed for each treatment: C50 and AMF50 under non-saline (0 mM NaCl) and saline (200 mM NaCl) conditions. These treatments were selected based on their pronounced morphological, nutrient, and metabolic differences. RNA quality and concentration were assessed using a NanoDrop™ 2000c spectrophotometer (Thermo Fisher Scientific, USA), with 10 µL collected for sequencing. Messenger RNA (mRNA) was purified from total RNA using poly-T oligo-attached magnetic beads, followed by fragmentation. First-strand cDNA was synthesized with random hexamer primers and reverse transcription, while second-strand cDNA synthesis used dUTP (for directional libraries) or dTTP (for non-directional libraries). Library preparation was performed using the NEBNext^®^ Ultra™ RNA Library Prep Kit for Illumina^®^ (NEB, USA), according to the manufacturer’s instructions. This included end repair, A-tailing, adapter ligation, size selection, USER enzyme digestion (for directional libraries), PCR amplification, and purification. Library quality was assessed using Qubit, real-time PCR, and an Agilent Bioanalyzer 2100 system. Sequencing was performed on an Illumina platform, generating 150 bp paired-end reads.

Clean reads were aligned to the reference genome (https://www.ncbi.nlm.nih.gov/datasets/genome/GCF_001683475.1/) using HISAT2 (v2.0.5). Gene expression levels were quantified using featureCounts (v1.5.0-p3), and differential expression analysis was performed with DESeq2, with significance thresholds set at a corrected p-value < 0.05 and |log2 (fold change)| > 1. KEGG pathway enrichment analysis was conducted using the clusterProfiler R package.

Functional annotation of differentially expressed genes was based on homology to annotated proteins in public databases (e.g., UniProt/TAIR), and gene functions are reported according to their closest characterized homologs.

### 2.10 Weighted gene co-expression network analysis

WGCNA was performed in R (v.4.4.1) using the WGCNA package (v.1.73) to identify gene modules associated with key traits under salinity stress and AMF inoculation. Gene expression data were preprocessed to exclude low-variance or missing values, then normalized using variance-stabilizing transformation (VST). A soft threshold (β = 31) was applied for scale-free topology (R² > 0.8). Genes with similar expression patterns were grouped into modules using a dynamic tree-cutting algorithm, with a minimum module size of 35 genes. Highly correlated modules (correlation > 0.75) were merged. Pearson’s correlation was used to assess the relationship between modules and traits, and a heat map was generated to highlight significant module-trait associations.

### 2.11 Statistical analysis

Statistical analyses were performed using R software (v.4.4.1). Outliers were removed using the boxplot method. Normality and homogeneity of variance were assessed with the Shapiro–Wilk and Levene’s tests, respectively. Data meeting these assumptions were analyzed using a two-way ANOVA followed by Tukey’s HSD test (p < 0.05). Data that did not meet these assumptions were analyzed using the Kruskal–Wallis test followed by Dunn’s *post hoc* test (p < 0.05). For each measured variable, *post hoc* comparisons were conducted across all treatment combinations, comparing all 12 groups simultaneously. Different lowercase letters in figures and tables indicate significant differences among all groups for a given variable at p < 0.05. The value of n represents biological replicates (independent plants). No technical replicates were included in this study.

## 3 Results

### 3.1 Effects of salinity and AMF inoculation on growth, Chl levels, and root colonization in quinoa

**Figure 1:**
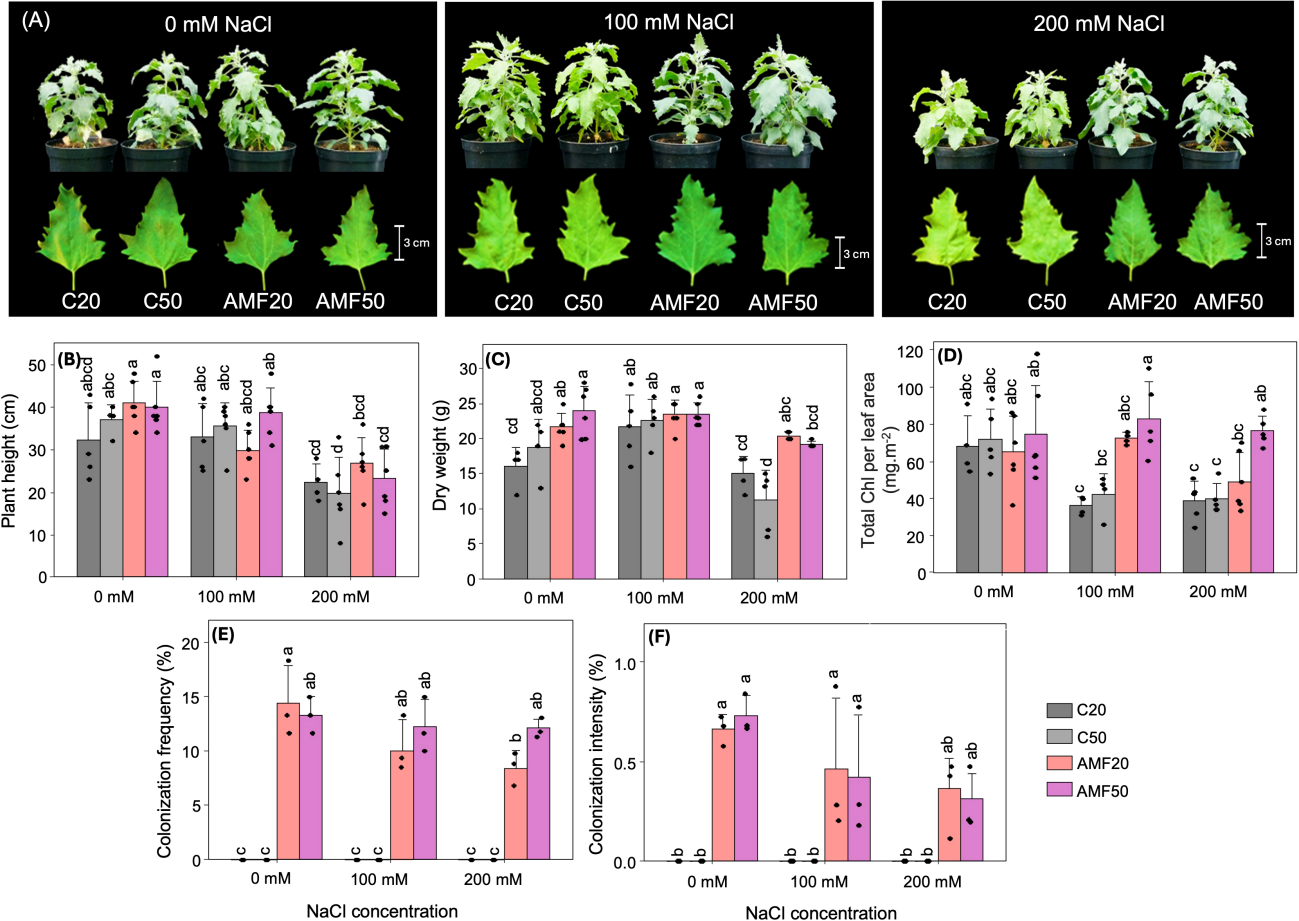
Growth responses, changes in total Chl concentration, and root colonization rates of arbuscular mycorrhizal fungi (AMF) in quinoa under different AMF treatments (C20 and C50: 20 g and 50 g sterilized inoculum; AMF20 and AMF50: 20 g and 50 g AMF inoculum) and salinity levels (0, 100, and 200 mM NaCl). **(A)** plants and leaf appearance. **(B)** plant height. **(C)** dry weight. **(D)** total Chl concentration. **(E)** colonization frequency. **(F)** colonization intensity. Data are presented as means + SD, n = 3-6. Dots represent individual biological replicates. Different lowercase letters indicate significant differences among all combinations of treatment and salinity (p < 0.05), based on Tukey’s HSD test (plant height, total Chl concentration) and Dunn’s test (dry weight, colonization frequency and intensity). Chl: chlorophyll.

The analysis of quinoa growth under varying salinity and AMF inoculation levels revealed distinct growth responses. As salinity increased, visible changes in leaf color were observed in non-inoculated plants (**Figure 1A**), which appeared more yellowish, especially at higher NaCl concentrations, suggesting stress-induced effects. In contrast, AMF-inoculated plants displayed a greener and healthier phenotype, which may be indicative of improved salinity tolerance associated with AMF (**Figure 1A**). Although AMF inoculation did not lead to significant increases in plant height or dry weight across all treatments, a slight increase in both parameters was observed in AMF-inoculated plants at high salinity (**Figures 1B, C**).

Consistent with these phenotypic observations, total Chl, Chl a, and Chl b were reduced by salinity in non-inoculated plants (**Figure 1D**, **Supplementary Figures 1A, B**). Under non-saline conditions, all treatments displayed similar levels of Chl a, b, and total Chl, indicating that AMF inoculation did not significantly affect Chl concentration in the absence of stress. However, under saline conditions, AMF-inoculated plants exhibited higher Chl levels than non-inoculated plants. Notably, AMF50 plants maintained higher total Chl concentrations under salinity stress, showing 2-fold and 1.9-fold levels at moderate and high salinity, respectively, compared to their corresponding non-inoculated controls. Chl a and b followed a similar pattern, suggesting a potential role of AMF in alleviating salinity-induced disruptions in Chl metabolism (**Figure 1D**, **Supplementary Figures 1A, B**).

All AMF-inoculated roots showed successful colonization, while those treated with sterilized AMF displayed no colonization (**Figures 1E, F**). Salinity treatments appeared to decrease colonization in AMF-inoculated plants, indicating a mild inhibitory effect on AMF colonization. In AMF-inoculated plants, colonization frequency ranged from 8.9% to 14.4%, and intensity ranged from 0.3% to 0.7%, with the highest values observed under control conditions, followed by the 100 mM NaCl treatment. Notably, there was no significant difference in colonization rates between AMF20 and AMF50 treatments (**Figures 1E, F**).

### 3.2 Effects of salinity and AMF inoculation on leaf ion concentrations

**Figure 2:**
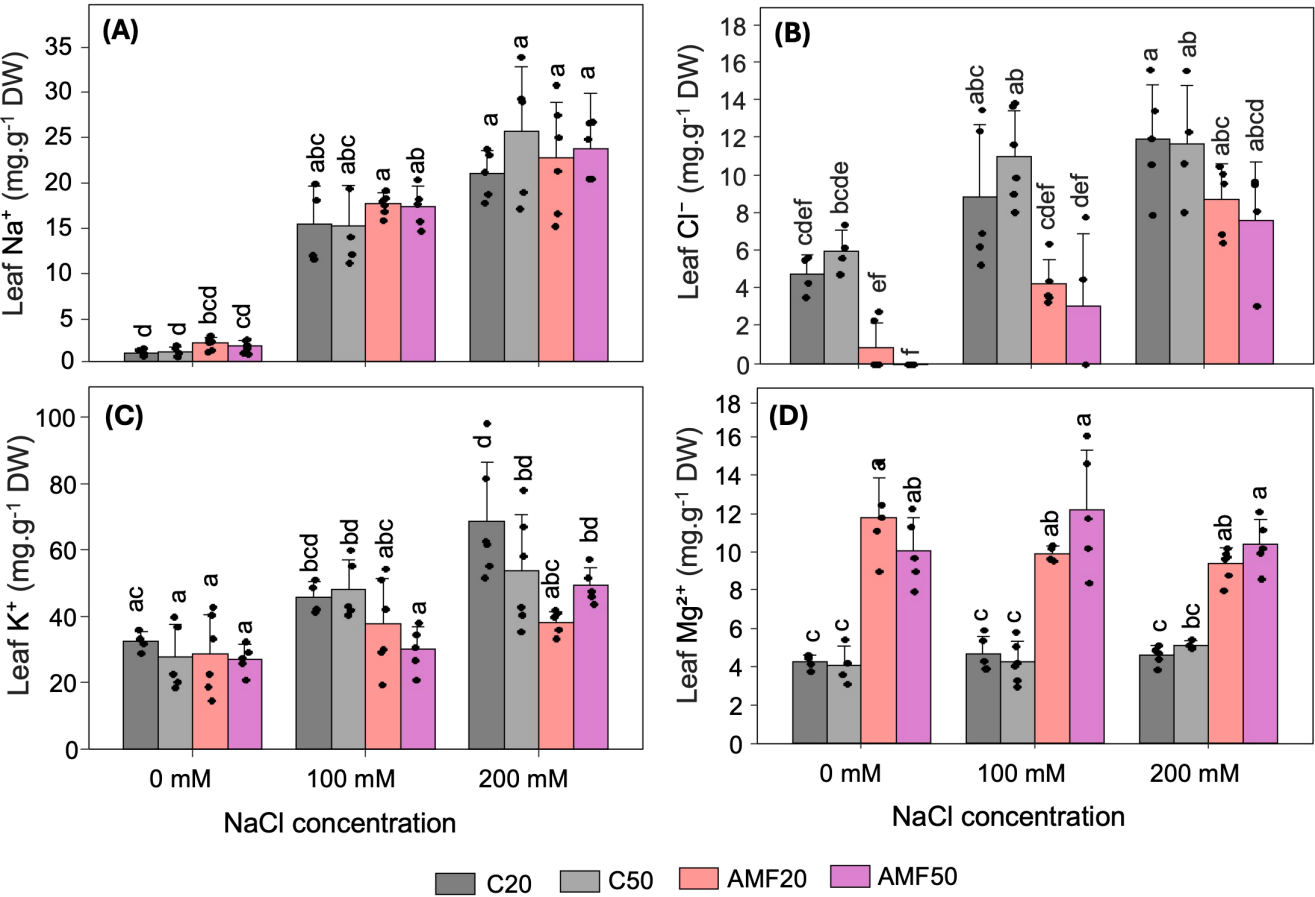
Macro- and micronutrient concentrations in quinoa leaves subjected to different arbuscular mycorrhizal fungi (AMF) treatments (C20 and C50: 20 g and 50 g sterilized inoculum; AMF20 and AMF50: 20 g and 50 g AMF inoculum) and salinity levels (0, 100, and 200 mM NaCl). **(A)** sodium. **(B)** chloride. **(C)** potassium. **(D)** magnesium. Data are presented as means + SD, n = 3-6. Dots represent individual biological replicates. Different lowercase letters indicate significant differences among all combinations of treatment and salinity (p < 0.05), based on Tukey’s HSD test (leaf Na^+^) and Dunn’s test (leaf Cl^-^, K^+^, Mg²^+^). DW: dry weight.

Na^+^ and Cl^-^ are the primary ions responsible for the toxic effects of salinity stress. Their concentrations increased in quinoa leaves under salinity in non-inoculated plants (**Figures 2A, B**). While AMF inoculation had no detectable effect on Na^+^ levels, a consistent tendency toward reduced Cl^-^ accumulation was observed in AMF-inoculated plants across all conditions. Under non-saline conditions, AMF inoculation reduced Cl^-^ levels to negligible values compared with the non-inoculated controls. At moderate salinity, Cl^-^ levels decreased to 0.5-fold in AMF20 plants compared with C20 plants and to 0.3-fold in AMF50 plants compared with C50 plants. Similarly, at high salinity, Cl^-^ concentrations decreased to 0.7-fold in both AMF20 and AMF50 treatments compared with their respective controls (**Figure 2B**). Interestingly, potassium (K^+^) levels also responded markedly to salinity (**Figure 2C**). In non-inoculated plants, K^+^ concentrations increased significantly under both moderate and high salinity. AMF inoculation slightly decreased leaf K^+^ concentrations under salinity stress, while it had no effect under non-saline conditions (**Figure 2C**). These findings suggest that salinity leads to the accumulation of both toxic ions (Na^+^, Cl^-^) and beneficial ions such as K^+^, which may help stabilize the plant’s internal ion distribution under stress. AMF symbiosis might modulate ion homeostasis by limiting Cl^-^ accumulation without significantly affecting the Na^+^/K^+^ balance. Salinity had no detectable effect on Mg²^+^ concentration in either non-inoculated or AMF-inoculated plants. However, across all treatments, the Mg²^+^ concentration was higher in AMF-inoculated plants than in non-inoculated plants, and this trend was maintained under salinity (**Figure 2D**).

### 3.3 Primary metabolic responses of quinoa to salinity and AMF inoculation

**Figure 3:**
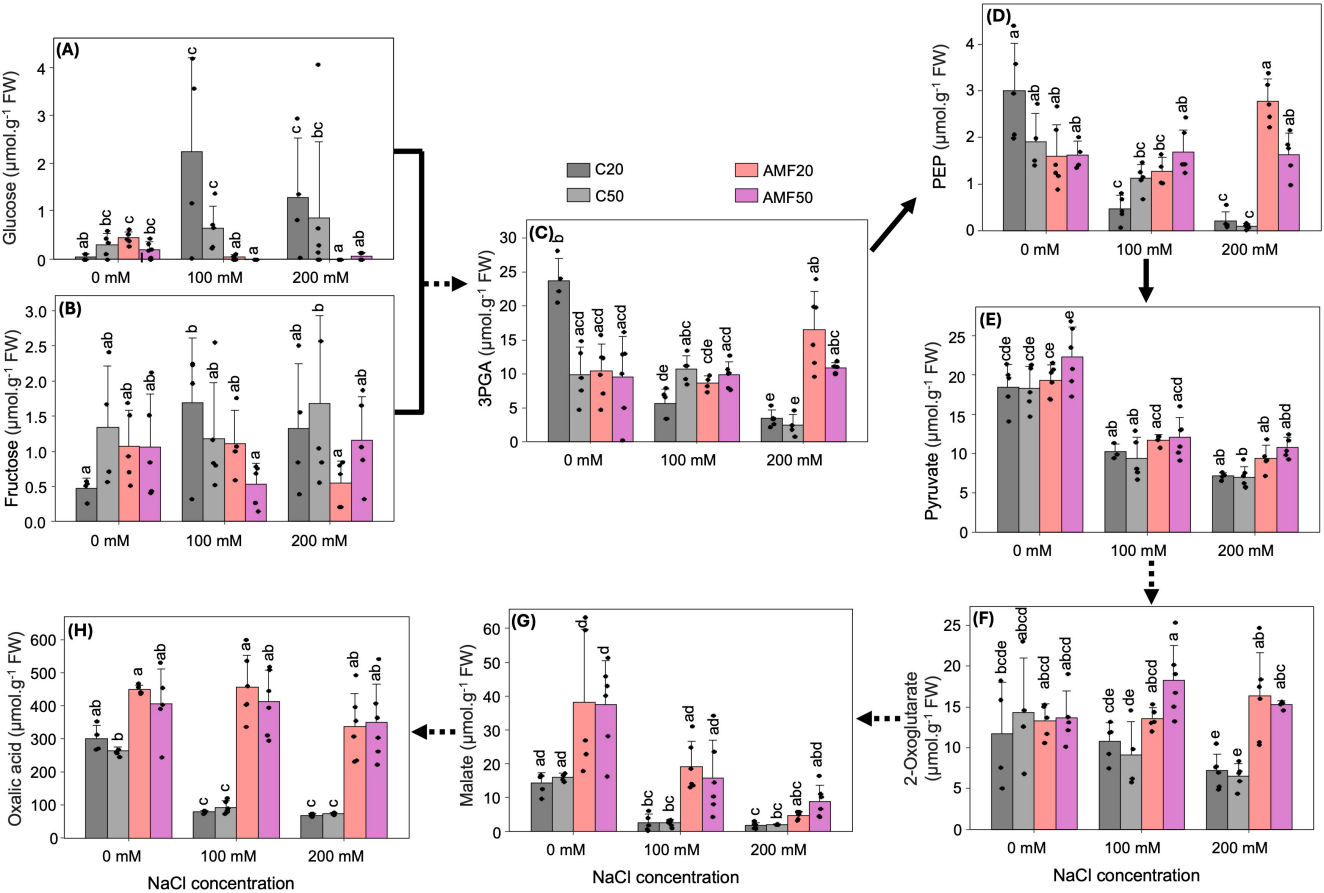
Primary metabolite concentrations in quinoa leaves subjected to different arbuscular mycorrhizal fungi (AMF) treatments (C20 and C50: 20 g and 50 g sterilized inoculum; AMF20 and AMF50: 20 g and 50 g AMF inoculum) and salinity levels (0, 100, and 200 mM NaCl). **(A, B)** sugars. **(C–E)** glycolytic intermediates. **(F–H)** organic acids. Data are presented as means + SD, n = 3-6. Dots represent individual biological replicates. Different lowercase letters indicate significant differences among all combinations of treatment and salinity (p < 0.05), based on Dunn’s test. Solid lines indicate direct biosynthesis, while dashed lines represent intermediate steps. FW, fresh weight; PEP, phosphoenolpyruvate; 3PGA, 3-phosphoglycerate.

Significant effects of salinity and AMF treatment were observed for glucose, 3-phosphoglycerate (3PGA), and phosphoenolpyruvate (PEP), while fructose and pyruvate showed only minor changes, with a slight decrease in fructose and a slight increase in pyruvate (**Figures 3A–E**). Non-inoculated plants showed a trend toward increasing glucose and fructose levels with rising salinity. While fructose levels were not significantly affected, glucose concentrations were markedly reduced by AMF treatment, becoming negligible under salinity stress (**Figures 3A, B**). Furthermore, salinity decreased levels of 3PGA, PEP, and pyruvate in non-inoculated plants, whereas AMF treatment largely maintained their concentrations, particularly 3PGA and PEP under high salinity (**Figures 3C–E**). Hence, 3PGA levels increased by approx. 4.9-fold in AMF20 and 4.4-fold in AMF50 plants relative to C20 and C50, respectively (**Figure 3C**). Similarly, PEP levels increased by more than 11-fold with AMF20 and AMF50 treatments under high salinity (**Figure 3D**). A decrease in sugars and an increase in glycolytic intermediates, including 3PGA, PEP, and pyruvate, could indicate alterations in central carbon metabolism associated with energy homeostasis and stress adaptation.

To better understand how AMF inoculation influences energy production and carbon flow in quinoa under salinity, key intermediates of the tricarboxylic acid (TCA) cycle were analyzed (**Figures 3F–H**, **Supplementary Table 2**). Results revealed significant effects of AMF inoculation and salinity on malate, oxalic acid, and 2-oxoglutarate contents, particularly under high salinity, whereas the remaining analyzed organic acids did not show significant changes (**Figures 3F–H**, **Supplementary Table 2**). The level of 2-oxoglutarate decreased slightly in non-inoculated plants exposed to salinity stress, with less than 0.5-fold at high salinity compared to the control (**Figure 3F**). In contrast, AMF-inoculated plants maintained constant levels of 2-oxoglutarate despite increasing salinity stress. In particular, AMF50 plants showed no significant decline at moderate and high salinity, compared to their controls (**Figure 3F**). In non-inoculated plants, malate levels decreased significantly with increasing salinity, showing reductions of approx. 0.2-fold and 0.1-fold at moderate and high salinity, respectively (**Figure 3G**). However, compared to non-inoculated plants, AMF-inoculated plants maintained higher malate across all salinity levels. The largest differences were observed under non-saline and moderate salinity, with malate levels approx. 2.7- and 2.3-fold higher in AMF20 and AMF50, respectively, under non-saline conditions, and 7.5- and 7.9-fold higher under moderate salinity (**Figure 3G**). Oxalic acid concentration followed a similar trend in non-inoculated plants, with a significant decline under salinity (**Figure 3H**). The results showed reductions of 0.3- and 0.4-fold at moderate and high salinity, respectively. Notably, AMF-inoculated plants maintained oxalic acid levels under salinity at levels comparable to unstressed plants. Under moderate salinity, oxalic acid levels increased by approx. 5.8-fold and 4.5-fold in AMF20 and AMF50, respectively, while under high salinity, increases of 4.9-fold and 4.8-fold were observed compared to their respective controls (**Figure 3H**). No significant differences were observed between AMF20 and AMF50 treatments across all conditions (**Figures 3F–H**).

Amino acids play vital roles in stress responses, including osmotic adjustment, antioxidant defense, and energy balance. Their accumulation under salinity provides insight into how quinoa adapts to salinity stress at the metabolic level. Among the 19 amino acids analyzed, a slight reduction in leaf concentrations of glutamate, isoleucine, and alanine was observed in non-inoculated plants under salinity stress (**Table 1**, **Supplementary Table 2**). In contrast, proline levels increased slightly in non-inoculated plants, with a significant increase observed in C50 plants, reaching approx. 2-fold at high salinity (**Table 1**). The increase in proline levels is a typical plant response to salinity stress, given its role in osmotic adjustment. AMF inoculation potentially contributed to improved salinity tolerance in stressed plants by increasing the levels of these amino acids, with the most notable enhancements observed in AMF50 plants. Hence, under moderate salinity, glutamate level increased by approx. 3.9-fold, proline by 1.8-fold, alanine by 3.3-fold, and isoleucine by 2.7-fold with AMF50 treatment compared to non-inoculated plants. At high salinity, glutamate levels increased by 4.6-fold, proline by 1.9-fold, alanine by 3.1-fold, and isoleucine by 2.3-fold (**Table 1**). Other amino acids, including GABA, histidine, and glutamine, were instead decreased by AMF inoculation, suggesting a shift toward preferential metabolic pathways under AMF treatment (**Supplementary Table 3**).

**Table 1:** Amino acid concentrations in quinoa leaves subjected to different arbuscular mycorrhizal fungi (AMF) treatments (C20 and C50: 20 g and 50 g sterilized inoculum; AMF20 and AMF50: 20 g and 50 g AMF inoculum) and salinity levels (0, 100, and 200 mM NaCl).

Amino acid (nmol.g^-1^ FW)	Treatment	0 mM	100 mM	200 mM
Glutamate	C20	2025 ± 448 ab	1357 ± 260 b	1631 ± 506 b
	C50	2230 ± 575 ab	1702 ± 625 b	1329 ± 735 b
	AMF20	2880 ± 710 ab	5976 ± 394 a	4146 ± 964 ab
	AMF50	4061 ± 873 a	6646 ± 590 a	6195 ± 303 a
Proline	C20	357 ± 112 def	507 ± 420 bcdef	548 ± 99 abcdef
	C50	308 ± 114 de	464 ± 126 cdef	596 ± 78 abcf
	AMF20	319 ± 97 de	672 ± 120 abc	779 ± 306 abc
	AMF50	231 ± 108 d	845 ± 317 ab	1138 ± 406 a
Alanine	C20	509 ± 94 bcde	304 ± 219 cde	289 ± 131 e
	C50	843 ± 239 abc	321 ± 164 de	241 ± 163 e
	AMF20	995 ± 260 bcde	853 ± 130 abc	438 ± 182 cde
	AMF50	992 ± 165 ab	1051 ± 425 a	743 ± 131 abcd
Isoleucine	C20	127 ± 38 cde	106 ± 79 cde	86 ± 27 e
	C50	199 ± 81 bcde	120 ± 57 de	117 ± 64 de
	AMF20	357 ± 87 a	203 ± 65 bcde	236 ± 68 abcd
	AMF50	263 ± 90 abc	320 ± 70 ab	267 ± 46 abc

Data are presented as means ± SD, n = 3–6 biological replicates. Different lowercase letters indicate significant differences among all combinations of treatment and salinity (p < 0.05), based on Tukey’s HSD test (alanine and isoleucine), and Dunn’s test (glutamate and proline). FW: fresh weight.

### 3.4 Effects of salinity and AMF inoculation on phenolic compound concentrations

To assess the antioxidative response of quinoa under salinity, 9 phenolic compounds were analyzed (**Supplementary Table 2**). These compounds were selected for their known role in mitigating oxidative damage during stress. Among the phenolic compounds analyzed in quinoa leaves, ferulic acid, benzoic acid, and p-coumaric acid showed a significant decrease with increasing salinity in sterilized plants (**Table 2**). Compared to non-inoculated plants, ferulic acid concentration increased by approx. 6.4-fold in AMF20 and by 4.6-fold in AMF50 plants at moderate salinity, and by approx. 4.8-fold and 6-fold at high salinity. Benzoic acid increased by approx. 2.3-fold and 2.2-fold at moderate salinity in AMF20 and AMF50 plants, respectively, and by approx. 3.5-fold and 3.3-fold at high salinity. Meanwhile, p-coumaric acid increased by 5.3-fold and 9.2-fold at moderate salinity and by 3.1-fold and 3.5-fold at high salinity with AMF20 and AMF50 treatments, respectively. AMF inoculation maintained the concentrations of ferulic acid, benzoic acid, and p-coumaric acid at levels similar to those observed under non-saline conditions, particularly at moderate salinity (**Table 2**).

**Table 2:** Phenolic acid concentrations in quinoa leaves subjected to different arbuscular mycorrhizal fungi (AMF) treatments (C20 and C50: 20 g and 50 g sterilized inoculum; AMF20 and AMF50: 20 g and 50 g AMF inoculum) and salinity levels (0, 100, and 200 mM NaCl).

Phenolic compound (nmol.g^-1^ FW)	Treatment	0 mM	100 mM	200 mM
Ferulic acid	C20	22.70 ± 3.10 bcd	5.62 ± 4.04 de	3.17 ± 2.08 e
	C50	24.68 ± 6.43 bc	9.02 ± 7.04 cde	3.86 ± 4.89 de
	AMF20	36.36 ± 5.58 ab	35.98 ± 10.55 ab	15.13 ± 3.60 cde
	AMF50	25.74 ± 5.25 abc	41.28 ± 11.36 a	23.06 ± 5.52 bc
Benzoic acid	C20	22.88 ± 3.45 ab	9.38 ± 4.06 bc	6.49 ± 2.71 c
	C50	23.94 ± 4.86 ab	9.51 ± 3.89 bc	7.71 ± 3.89 bc
	AMF20	19.07 ± 8.09 abc	21.23 ± 9.09 abc	22.25 ± 7.40 ab
	AMF50	24.93 ± 10.56 ab	20.59 ± 7.94 abc	24.86 ± 6.40 a
p-Coumaric acid	C20	4.64 ± 4.41 abc	2.26 ± 2.40 c	1.80 ± 0.92 c
	C50	2.13 ± 0.95 bc	1.15 ± 1.13 c	1.73 ± 0.77 c
	AMF20	11.27 ± 4.24 a	12.03 ± 3.82 a	5.51 ± 0.93 abc
	AMF50	8.10 ± 2.19 ab	10.55 ± 4.14 a	6.08 ± 2.88 abc

Data are presented as means ± SD, n = 3–6 biological replicates. Different lowercase letters indicate significant differences among all combinations of treatment and salinity (p < 0.05), based on Tukey’s HSD test (ferulic acid, benzoic acid), and Dunn’s test (p-coumaric acid). FW: fresh weight.

### 3.5 Multivariate analysis of metabolic and nutrient profiles under salinity and AMF inoculation

**Figure 4:**
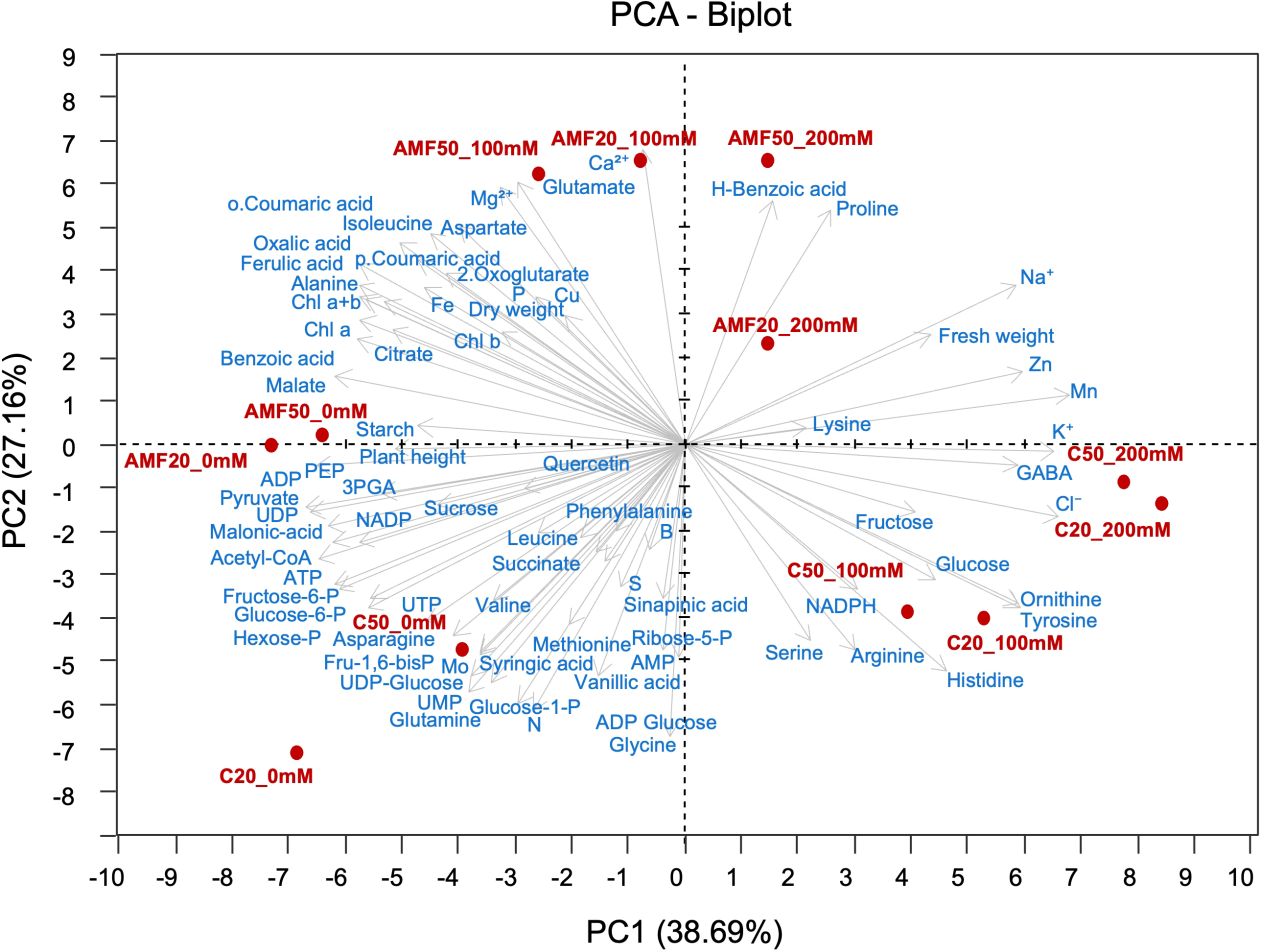
Principal component analysis (PCA) biplot of metabolic and nutrient profiles in quinoa leaves subjected to different arbuscular mycorrhizal fungi (AMF) treatments (C20 and C50: 20 g and 50 g sterilized inoculum; AMF20 and AMF50: 20 g and 50 g AMF inoculum) and salinity levels (0, 100, and 200 mM NaCl). Treatments are labeled in red (AMF treatments_salinity level), while variables are labeled in blue. PEP, phosphoenolpyruvate; 3PGA, 3-phosphoglycerate; Chl, chlorophyll.

The PCA was performed using all measured parameters in this study (see **Supplementary Table 1**) to avoid bias and provide a comprehensive overview of plant responses. A clear separation among treatment groups was observed, highlighting distinct metabolic patterns influenced by salinity and AMF inoculation (**Figure 4**). Along PC1, a gradient reflecting the impact of salinity was observed: non-stressed plants (0 mM NaCl), regardless of AMF inoculation, clustered on the negative side and were associated with sugar precursors, energy-related metabolites (e.g., ATP, UTP), and several organic, amino, and phenolic acids, indicating an active and balanced metabolism. In contrast, non-inoculated plants under salinity stress showed greater dispersion, indicating weaker, less coordinated metabolic adaptation. Meanwhile, the upper region of PC2 was defined by AMF-inoculated plants under salinity, which clustered with proline, glutamate, phenolic acids, Chl, and nutrients, including calcium (Ca²^+^), magnesium (Mg²^+^), and zinc, highlighting a coordinated metabolic response likely contributing to improved stress tolerance (**Figure 4**). These results collectively suggest a potential role of AMF in modulating plant metabolism under salinity, enhancing the accumulation of protective compounds and maintaining nutrient balance, whereas the absence of viable AMF is associated with a more stress-prone metabolic profile.

### 3.6 Transcriptomic responses to salinity and AMF inoculation

To uncover the molecular mechanisms underlying quinoa’s response to salinity stress and AMF inoculation, transcriptomic profiling was conducted to compare non-inoculated (C50) and AMF-inoculated (AMF50) plants under non-saline (0 mM NaCl) and highly saline (200 mM NaCl) conditions.

**Figure 5:**
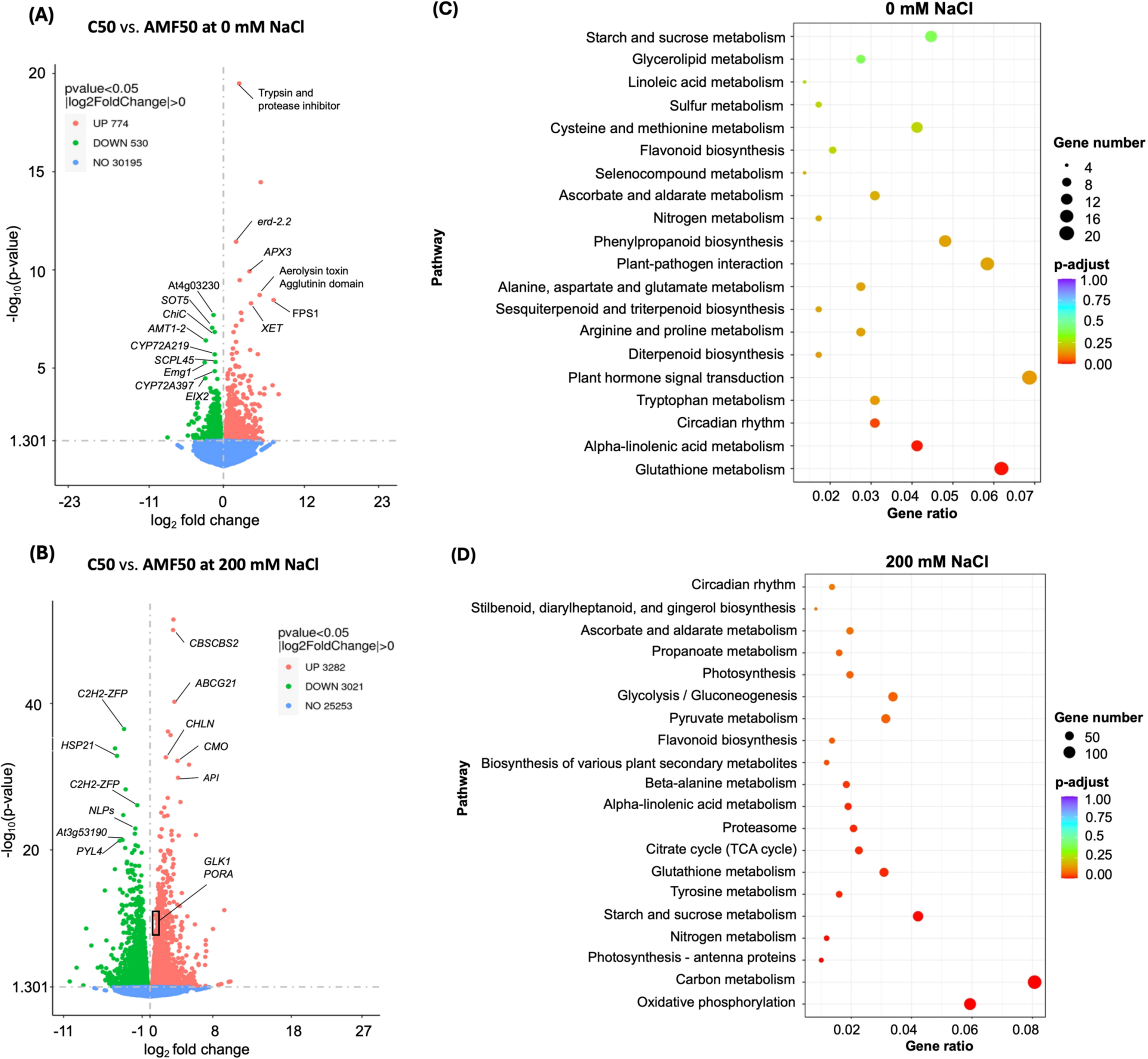
Gene expression analysis comparing quinoa plants inoculated with 50 g of arbuscular mycorrhizal fungi (AMF) inoculum under control (0 mM NaCl) and salinity stress (200 mM NaCl) conditions. **(A, B)** volcano plots displaying differentially expressed genes, categorized as upregulated or downregulated (p < 0.05, |log_2_ fold change| > 1). **(C, D)** KEGG enrichment of the top 20 differentially expressed genes under 0 and 200 mM NaCl, ranked by gene ratio and adjusted p-value (padj).

Differential gene expression analysis revealed substantial transcriptional modulation in response to salinity. Under salinity, 6, 303 genes were differentially expressed, compared to only 1, 304 DEGs under non-saline conditions (**Figures 5A, B**). Salinity stress predominantly induced gene upregulation, with 3, 282 genes upregulated and 3, 021 downregulated, compared with the non-saline condition, where only 774 genes were upregulated and 530 downregulated. KEGG pathway enrichment analysis revealed distinct transcriptional responses among conditions (**Figures 5C, D**). Under non-saline conditions, AMF inoculation had a modest impact, with enrichment in pathways related to glutathione metabolism, alpha-linolenic acid metabolism, and circadian rhythm modulation (**Figure 5C**). However, under salinity stress, AMF-inoculated plants exhibited significant upregulation of carbon metabolism, starch and sucrose metabolism, TCA cycle, and photosynthesis-associated proteins, suggesting a metabolic adaptation associated with AMF inoculation. Oxidative phosphorylation, N metabolism, and proteasome activity were also among the most enriched pathways, alongside notable shifts in secondary metabolic pathways, further supporting a potential role of AMF in modulating stress responses at the molecular level (**Figure 5D**). Several genes involved in these enriched metabolic pathways were strongly upregulated under salinity (**Figure 5B**). These genes include *CBSCBS2* (*Arabidopsis thaliana* homolog), involved in carbon metabolism; *CHLN* (*Solanum lycopersicum* homolog), encoding an iron chelatase subunit crucial for Chl biosynthesis; and *CMO* (*Solanum lycopersicum* homolog; choline monooxygenase), linked to glycine betaine biosynthesis and osmoprotection. Notably, *GLK1* (*Arabidopsis thaliana* homolog), a transcriptional regulator of the photosynthetic machinery, was significantly upregulated, with a log_2_ fold change of +1.05 (p = 8.18 × 10^-12^). Similarly, *PORA* (*Cucumis sativus* homolog), which encodes a key enzyme involved in protochlorophyllide reduction, also showed upregulation with a log_2_ fold change of +1 (p = 1.23 × 10^-7^). These findings are consistent with a potential role of AMF in maintaining photosynthetic efficiency under saline conditions (**Figure 5B**).

### 3.7 Co-expression network analysis of gene modules associated with metabolites and nutrients under salinity and AMF inoculation

**Figure 6:**
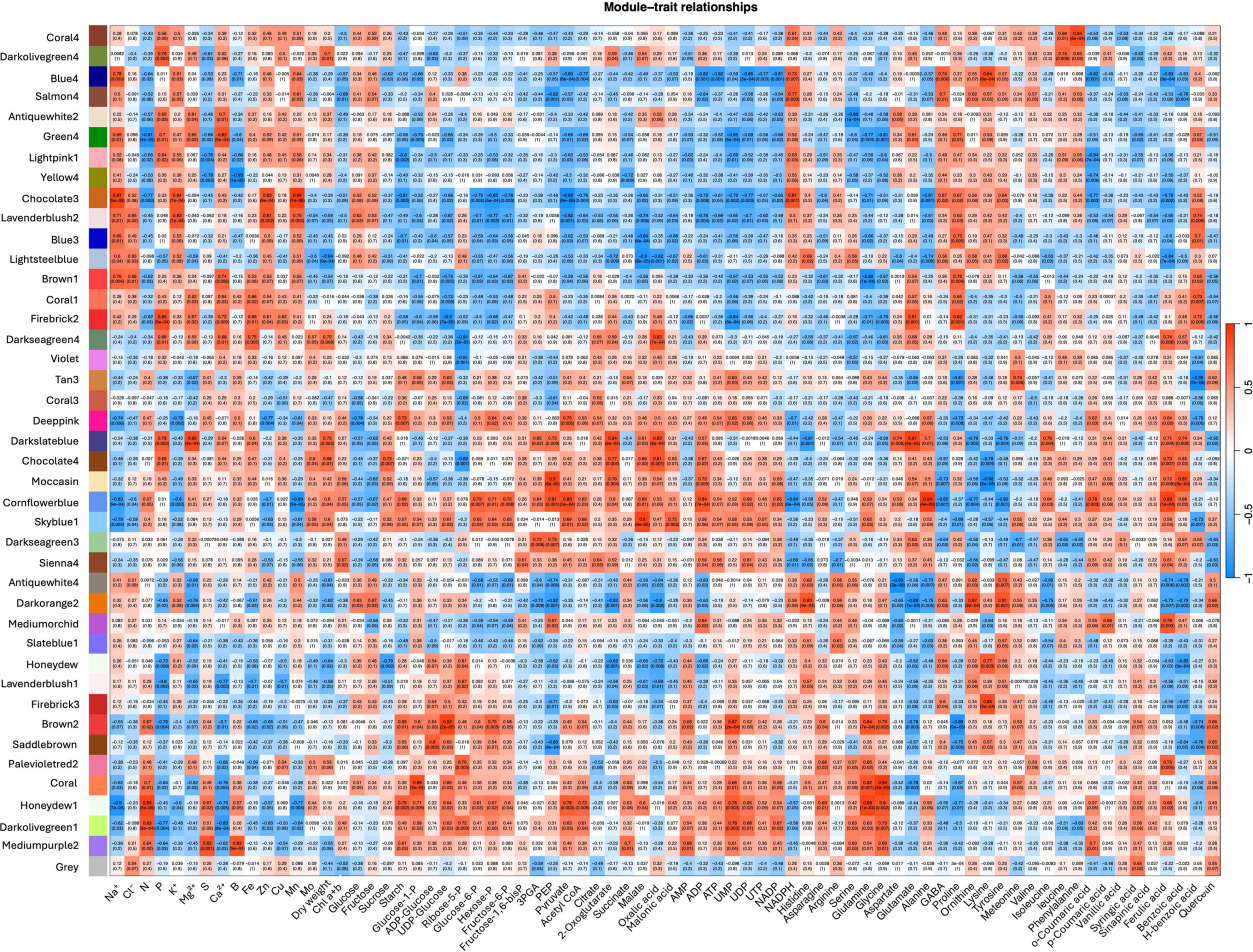
Heatmap depicting the correlation between eigengene modules and key metabolic traits derived from weighted gene co-expression network analysis (WGCNA) of differentially expressed genes in quinoa subjected to AMF treatment and salinity stress. Correlation index and p-values are indicated in parentheses. Eigengene modules were derived using a soft-thresholding power of 31, and 23,016 genes from RNA sequencing (12 samples) were analyzed. PEP, phosphoenolpyruvate; 3PGA, 3-phosphoglycerate; Chl, chlorophyll.

To dissect the molecular basis of AMF-mediated salinity tolerance in quinoa, WGCNA was employed to identify gene clusters (modules) associated with key metabolites and nutrients. This approach enabled a global analysis of transcriptional networks, revealing co-expression patterns rather than isolated gene responses. Of 43, 952 genes obtained from RNA sequencing across 12 samples, stringent filtering retained 23, 016 high-confidence genes, grouped into 42 distinct co-expression modules (**Figure 6**, **Supplementary Table 4**). The largest module (chocolate3) contained 3, 283 genes, while the smallest (slateblue1) comprised only 52 differentially expressed genes (DEGs) (**Supplementary Table 4**).

Gene expression profiling revealed stark contrasts between AMF-treated and control plants under salinity stress (**Figure 6**). Notably, seven modules (darkslateblue, cornflowerblue, firebrick2, darkseagreen3, sienna4, moccasin, and coral1) were significantly upregulated in AMF-inoculated plants exposed to salinity stress (**Supplementary Figure 4**). Among these, darkslateblue and cornflowerblue showed strong positive correlations (p < 0.05) with a greater number of parameters, including dry weight and metabolites involved in carbon metabolism, osmotic adjustment, and nutrient homeostasis. In particular, the darkslateblue module was positively correlated with dry weight and concentrations of PEP, 3PGA, 2-oxoglutarate, malate, oxalic acid, ADP, alanine, glutamate, isoleucine, aspartate, ferulic acid, benzoic acid, and the nutrient elements Mg²^+^, P, and Fe (**Figure 6**). Meanwhile, the cornflowerblue module was positively correlated with dry weight, Chl content, and a wide range of metabolites, including PEP, pyruvate, 3PGA, starch, glucose-1-P, hexose-P, fructose-6-P, acetyl-CoA, 2-oxoglutarate, malate, ADP, UDP, NADP, alanine, isoleucine, ferulic acid, benzoic acid, o-coumaric acid, and N concentration. These associations indicate coordinated transcriptional responses under AMF treatment, reflecting co-expression patterns related to carbon allocation, energy metabolism, and nutrient acquisition under salinity, and are consistent with improved stress resilience in quinoa.

A closer analysis of AMF-associated modules highlighted several genes with known roles in metabolic pathways linked to stress adaptation (**Table 3**). Genes within the darkslateblue module, including *PHS2* (carbohydrate phosphorylase), *PPD* (phosphate dikinase), *TD1* (pyridoxal-phosphate-dependent enzyme), and *MASY*_*GOSHI* (malate synthase), are known to be involved in the biosynthesis of PEP, isoleucine, and oxalic acid, key intermediates in energy metabolism and osmoprotection. The *MHX* gene (Mg²^+^/H^+^ exchanger), also present in this module, is associated with Mg²^+^ homeostasis and may contribute to ionic balance under salinity stress. Similarly, the cornflowerblue module contained genes such as *PK* (pyruvate kinase) and *PDHB* (pyruvate dehydrogenase), which are involved in the pyruvate-2-oxoglutarate metabolic pathway, indicating a link between this module and central carbon metabolism (**Table 3**).

**Table 3:** Summary of module eigengenes, correlated metabolites and nutrients, and regulated genes identified by WGCNA in quinoa subjected to arbuscular mycorrhizal fungi (AMF) treatment and salinity stress.

Module eigengene	Metabolite / nutrient	Regulated genes (annotation source)	Function
darkslateblue (1546 genes)	PEP	*PHS2* (*Arabidopsis thaliana*)	Starch phosphorylase
	PEP	*PPD* (*Mesembryanthemum crystallinum*)	Pyruvate phosphate dikinase
	Isoleucine	*TD1* (*Arabidopsis thaliana*, *Solanum lycopersicum*)	Threonine deaminase
	Oxalic acid	*MASY_GOSHI* (*Gossypium hirsutum*)	Malate synthase (*MS*)
	Mg²^+^	*MHX* (*Arabidopsis thaliana*)	Mg²^+^/H^+^ exchanger;
cornflowerblue (1921 genes)	Pyruvate	*PK* (Solanum tuberosum)	Pyruvate kinase
	2-Oxoglutarate	*PDHB* (*Arabidopsis thaliana*)	Pyruvate dehydrogenase
darkorange2 (1103 genes)	Glucose	*AMY2* (*Arabidopsis thaliana*)	Alpha-amylase; catalyzes starch degradation
	Fructose	*SDH* (*Arabidopsis thaliana*)	Sorbitol dehydrogenase; converts sorbitol to fructose
	Malate, pyruvate	*MAON_SOLTU* (*Solanum tuberosum*)	AD-dependent malic enzyme (*ME*)
	2-Oxoglutarate	*At4g26910* (*Arabidopsis thaliana*)	2-oxoglutarate dehydrogenase
	Glutamate	*At5g26710* (*Arabidopsis thaliana*)	Glutamate–tRNA ligase
	Isoleucine	*BCE2* (*Arabidopsis thaliana*)	Catalyzes isoleucine degradation
	Isoleucine	*At5g09300* (*Arabidopsis thaliana*)	Catalyzes isoleucine degradation

Eigengene modules were derived using a soft-thresholding power of 31, and 23,016 genes from RNA sequencing (12 samples) were analyzed.

Conversely, eight modules (darkorange2, brown2, antiquewhite4, salmon4, firebrick3, tan3, honeydew, and saddlebrown) were significantly downregulated in AMF-inoculated plants compared to non-inoculated controls, suggesting potential association with reduced stress-related metabolic disruptions (**Supplementary Figure 4**). Among these, darkorange2 and antiquewhite4 showed negative correlations with the largest number of parameters (p < 0.05) including dry weight and several key metabolites, including glucose-6-P, fructose-6-P, hexose-P, PEP, 3PGA, ADP, aspartate, glutamate, alanine, isoleucine, malate, 2-oxoglutarate, oxalic acid, ferulic acid, benzoic acid, as well as Mg²^+^, P, and Fe (**Figure 6**). This pattern suggests that these modules may be associated with metabolic processes linked to the turnover or depletion of these compounds.

Further analysis of the darkorange2 module identified genes such as *AMY2* (alpha amylase), *SDH* (sorbitol dehydrogenase), *MAON*_*SOLTU* (malic enzyme), and quinoa homologs of Arabidopsis genes *At4g26910* (a component of the 2-oxoglutarate dehydrogenase complex involved in TCA cycle decarboxylation), *At5g26710* (glutamate–tRNA ligase involved in glutamate utilization), and *At5g09300* (2-oxoisovalerate dehydrogenase, involved in isoleucine degradation), as well as *BCE2* (2-oxoacid dehydrogenase acyltransferase involved in isoleucine degradation). These genes are associated with pathways related to soluble sugar metabolism, PEP turnover, and amino acid metabolism (**Table 3**). In contrast, the antiquewhite4 module lacked genes directly linked to metabolic pathways, suggesting it may be associated with broader regulatory processes that indirectly influence metabolism (**Figure 6**).

## 4 Discussion

Arbuscular mycorrhizal symbiosis is a well-documented strategy for enhancing plant resilience to abiotic stresses, including salinity (Evelin et al., 2019). However, despite the beneficial effects of AMF being extensively studied in salt-sensitive species, the role of AMF in salt-tolerant crops remains less explored. In quinoa, a highly salt-tolerant pseudocereal, existing research has primarily focused on morphological and physiological traits, such as changes in leaf water potential, stomatal conductance, and Chl fluorescence (Toubali and Meddich, 2023; Toubali et al., 2023), leaving a critical gap in understanding the mechanisms that govern metabolic adjustments, nutrient acquisition, and transcriptional regulation in AMF-associated quinoa under salinity stress. Here, we provide a multi-dimensional analysis integrating metabolic, nutrient, and transcriptomic data to understand AMF-associated salinity tolerance in quinoa. By examining key metabolites, nutrient uptake patterns, and gene expression networks, our findings suggest that AMF may contribute to quinoa’s early adaptive responses to salinity by influencing ion homeostasis, carbon metabolism, and osmoprotectant accumulation.

### 4.1 AMF colonization contributes to growth and Cl^-^ restriction under salinity

Salinity negatively impacted AMF colonization rates in quinoa (**Figures 1E, F**), consistent with previous observations reported by Rivero et al. (2018) and Ait-El-Mokhtar et al. (2020). While mycorrhizal colonization was confirmed in this study, it occurred at low levels, aligning with earlier reports of minimal to no root colonization of quinoa when inoculated with AMF. For example, Kellogg et al. (2021) observed colonization rates ranging from 0% to 3% across multiple quinoa genotypes, and Urcelay et al. (2011) reported negligible or no mycorrhizal colonization. Moreover, several studies have classified quinoa as “inconsistently mycorrhizal” due to the inconsistent presence of the symbiosis across varying conditions (Brundrett, 2017; Cosme et al., 2018; Kellogg et al., 2021). Given these low colonization levels, the extent to which a fully functional symbiosis was established in our study may be questioned. Interestingly, AMF symbiosis was associated with measurable benefits under salinity stress, with inoculated plants displaying a greener phenotype and modest growth improvements at high salinity (**Figures 1A–C**). Similarly, previous studies have reported AMF-mediated biomass enhancement in quinoa under saline conditions (Toubali and Meddich, 2023; Toubali et al., 2023). In line with this, Kellogg et al. (2021) reported increased leaf greenness in quinoa cultivars despite the absence of significant growth responses under non-saline conditions, suggesting that AMF-induced physiological improvements may occur independently of significant growth responses.

Taken together, low colonization does not necessarily correlate with reduced growth or stress adaptation. Previous studies in quinoa have reported physiological or growth responses despite minimal colonization, without clearly identifying the underlying mechanisms (Klironomos, 2003; Kellogg et al., 2021). Our results suggest that AMF-associated stress adaptation may involve changes in gene expression and signaling pathways, leading to downstream metabolic alterations that support stress tolerance.

In the present study, no significant difference in colonization rates was observed between the AMF20 and AMF50 treatments, indicating that increasing inoculum concentration had a limited effect, likely due to quinoa’s weak compatibility with AMF. This also implies that the low inoculum dose (20 g) may already achieve the maximum colonization possible under the given conditions.

Leaf ion accumulation provides key insights into plant stress responses. As expected, quinoa accumulated high levels of Na^+^ and Cl^-^ with increasing salinity (**Figures 2A, B**), confirming substantial ionic stress. Interestingly, while AMF did not affect Na^+^ uptake, it suppressed Cl^-^ accumulation, suggesting that the beneficial effect of AMF symbiosis possibly relied more on reduced net Cl^-^ uptake rather than influencing overall sodium homeostasis. One plausible mechanism is that AMF may contribute to enhanced Cl^-^ sequestration in root vacuoles by modulating the expression or activity of Cl^-^ transporters, thereby limiting Cl^-^ translocation to leaves and preventing toxic accumulation (Evelin et al., 2019). However, this mechanism can only be confirmed through direct measurements of Cl^-^ distribution in root tissues and analysis of Cl^-^ transporter expression, which were beyond the scope of the present study. Previous studies have demonstrated the role of AMF in mitigating Cl^-^ toxicity under salinity (Evelin et al., 2009; Zuccarini and Okurowska, 2008).

Interestingly, potassium (K^+^) also responded markedly to salinity stress. In non-inoculated plants, K^+^ levels significantly increased under both moderate and high salinity, likely as a compensatory response to maintain ionic balance and counteract Na^+^ toxicity (**Figure 2C**). In contrast, AMF inoculation slightly reduced leaf K^+^ concentrations under salinity, contrary to the common trend of AMF enhancing K^+^ uptake. However, similar decreases in shoot K^+^ have been reported in AMF-inoculated plants under salinity stress (Wang et al., 2021), suggesting that AMF may alter K^+^ transport or distribution. A decrease in shoot K^+^ may reflect a lower requirement for K^+^ accumulation in AMF-inoculated plants due to their improved ionic and metabolic regulation.

### 4.2 Metabolic alteration by AMF sustains Chl biosynthesis under salinity

Salinity-induced stress progressively reduced Chl concentrations in non-inoculated plants (**Figure 1D**, **Supplementary Figures 1A, B**), a widely observed phenomenon attributed to osmotic and oxidative damage, ionic imbalances, and disrupted Chl biosynthesis pathways. Key mechanisms include the suppression of Chl biosynthetic enzymes, increased activity of Chl-degrading enzymes (e.g., chlorophyllase), and impaired uptake of essential nutrients for Chl production, such as Fe, N, and Mg²^+^ (Evelin et al., 2019). In contrast, AMF-inoculated plants maintained significantly higher Chl levels under salinity stress than non-inoculated ones, consistent with previously reported results (Toubali and Meddich, 2023; Toubali et al., 2023). Transcriptomic analyses further revealed that *GLK1* and *PORA*, two key regulators of Chl biosynthesis and photosynthetic activity (**Figure 5B**) maintained higher expression levels in AMF-inoculated plants under salinity. *GLK1* encodes a transcription factor that promotes Chl production and photosystem development (Waters et al., 2009), while *PORA* is critical for converting protochlorophyllide to chlorophyllide, a key step in Chl biosynthesis (Paddock et al., 2012).

N, Mg²^+^, and Fe are fundamental elements for Chl structure and biosynthesis (Rengel et al., 2023). While leaf N concentrations significantly decreased with increasing salinity, and Mg²^+^ levels remained unaffected, their overall content in quinoa leaves remained high (**Figure 2D**, **Supplementary Figure 2A**), suggesting that these elements were largely available in the soil. Given the critical role of these nutrients in Chl biosynthesis, two possibilities can be proposed: First, these nutrients may not significantly contribute to the observed decrease in Chl content in non-inoculated plants under salinity, and second, salinity may impair their utilization efficiency and distribution within the plant rather than their uptake, thereby reducing Chl biosynthesis. AMF had minimal influence on N levels but consistently supplied Mg²^+^ under all conditions (**Figure 2D**, **Supplementary Figure 2A**), suggesting a role in enhancing the internal allocation and utilization of these nutrients. Supporting this, KEGG analysis revealed enhanced N metabolism pathways (**Figure 5D**). Additionally, the *MHX* gene was part of the darkslateblue module identified by WGCNA, in which genes showed higher eigengene expression in AMF-inoculated plants than in non-inoculated plants (**Table 3**, **Supplementary Figure 4**) and were positively correlated with Mg²^+^ concentrations (p > 0.05) (**Figure 6**). *MHX* encodes a Mg²^+^/H^+^ exchanger involved in maintaining Mg²^+^ homeostasis by regulating its distribution between the vacuole and the cytosol (David-Assael et al., 2005). Salinity significantly reduced Fe availability (**Supplementary Figure 2D**), likely due to decreased solubility and mobility in saline soils (Grattan and Grieve, 1999); however, Fe concentrations remained above the critical threshold of 50 ppm (Rengel et al., 2023). This suggests that in the present study, AMF-associated enhancement of Fe uptake does not play a major role in maintaining Chl levels under salinity stress.

Beyond nutrient availability, metabolic adjustments likely played a crucial role in sustaining Chl biosynthesis under salinity. Salinity stress reduced key metabolic intermediates involved in Chl biosynthesis, including 3PGA, PEP, pyruvate, malate, oxalic acid, 2-oxoglutarate, and glutamate, while increasing glucose and fructose levels (**Figure 3**, **Table 1**). The accumulation of glucose and fructose is a typical stress response and helps osmotic adjustment (Evelin et al., 2019). However, AMF inoculation prevented decreases in 3PGA, PEP, oxalic acid, 2-oxoglutarate, and glutamate under high salinity, while simultaneously reducing glucose and fructose concentrations, suggesting that AMF may contribute to sustaining metabolic activity in the shoot under salinity stress.

WGCNA identified the darkorange2 module, which is less expressed in AMF-inoculated plants than in non-inoculated plants (**Supplementary Figure 4**) and showed a positive correlation with glucose and fructose (**Figure 6**). Within this module, two genes were related to soluble sugar metabolism (*AMY2* and *SDH*) (**Table 3**). *AMY2* is responsible for starch degradation into glucose and maltose (Ju et al., 2019), while *SDH* catalyzes the oxidation of sorbitol to fructose (Ohta et al., 2005). Consistent with the functions of these genes, the reduced expression of the darkorange2 module in AMF-inoculated plants may indicate a lower demand for soluble sugar accumulation and greater metabolic flux toward central metabolism. Unlike previous studies, in which AMF increased soluble sugars under salinity (Chen et al., 2024; Singh et al., 2024), our findings indicate that AMF may instead contribute to redirecting carbon metabolism toward Chl biosynthesis and stress-adaptive pathways. This hypothesis is further supported by KEGG analysis, which identified significant enrichment of pathways related to carbon metabolism, photosynthesis, starch/sucrose metabolism, and the citrate cycle in AMF-inoculated plants (**Figures 5C, D**). Several genes with known roles in Chl biosynthesis pathways were co-expressed within WGCNA modules. Within the cornflowerblue module, co-expressed genes with higher expression levels in AMF-inoculated plants were positively correlated with pyruvate and 2-oxoglutarate, precursors of Chl biosynthesis and N assimilation (**Supplementary Figure 4**, **Table 3**, **Figure 6**). Notably, *PK* and *PDHB* are known to be involved in these pathways. *PK* catalyzes the conversion of PEP to pyruvate in glycolysis (UniProt, 2025), while *PDHB* encodes a chloroplastic pyruvate dehydrogenase complex, responsible for converting pyruvate into acetyl-CoA, a key substrate for the TCA cycle and Chl biosynthesis (Bhandary and Aguan, 2015).

### 4.3 AMF contributes to alleviating osmotic imbalance and oxidative stress under salinity

While AMF-associated metabolic shifts favored Chl biosynthesis, additional osmoprotectant accumulation and antioxidant defense pathways were also activated. Organic acids, known for their role as osmolytes, play a crucial role in mitigating abiotic stresses (Evelin et al., 2019). In this study, organic acids, including oxalic acid, 2-oxoglutarate, and malate, which were depleted under salinity stress, accumulated significantly in AMF-inoculated plants (**Figures 3F–H**). This aligns with previous findings demonstrating AMF-enhanced organic acid biosynthesis in salinity-stressed crops, including maize (Sheng et al., 2011) and peanut (Liu et al., 2023). Among these organic acids, oxalic acid was particularly abundant in AMF-inoculated plants. Oxalic acid plays a dual role: enhancing antioxidant enzyme activity (e.g., superoxide dismutase (SOD) and peroxidase (POD)) to mitigate ROS accumulation and acting as a chelator, improving the availability and uptake of nutrients such as Mg²^+^ and Ca²^+^ (**Figure 3H**, **Supplementary Figure 2C**) (Ahmad et al., 2019). Additionally, oxalic acid biosynthesis is closely linked to ascorbate metabolism, suggesting that increased oxalic acid levels may reflect elevated ascorbate concentrations, further strengthening the plant’s antioxidant defenses. Similarly, 2-oxoglutarate, a precursor for glutamate biosynthesis, likely supports the synthesis of stress-protective amino acids such as proline, while malate contributes to osmotic adjustment and photosynthetic regulation by increasing Chl content and stomatal opening, thereby sustaining growth under salinity (**Figures 3F, G**) (Ahmad et al., 2019).

Amino acids serve as osmoprotectants, metabolic precursors, and stress markers (Evelin et al., 2019). In this study, several amino acids, including glutamate, proline, alanine, and isoleucine, were higher in AMF-inoculated plants under salinity (**Table 1**). Notably, proline, a well-established osmoprotectant and stress marker (Evelin et al., 2019), accumulated in both inoculated and non-inoculated plants, with slightly higher levels in inoculated plants, suggesting an enhanced adaptive response facilitated by AMF. Alongside alanine and isoleucine, proline helps maintain osmotic balance, ensuring cellular hydration and improved stress tolerance. Interestingly, while these protective amino acids increased, others, such as GABA (Gamma-aminobutyric acid), glutamine, and histidine, were reduced under salinity stress, with an even greater decline in AMF-inoculated plants (**Supplementary Table 3**). This may reflect a preferential allocation of metabolic resources toward specific stress-adaptive pathways in AMF-inoculated plants, such as Chl and proline biosynthesis. Similar AMF-associated increases in amino acid levels under salinity have been reported in seepweed (Jia et al., 2024).

In addition to primary metabolites, phenolic compounds play a crucial role in plant stress adaptation by acting as non-enzymatic antioxidants that scavenge ROS and neutralize oxidative damage (Benaffari et al., 2022). While salinity significantly reduced the biosynthesis of phenolic acids such as ferulic, benzoic, and p-coumaric acid, AMF-inoculated plants exhibited higher accumulation of these compounds (**Table 2**). The enrichment of phenylpropanoid-derived antioxidants aligns with a previous study in quinoa (Benaffari et al., 2022), where AMF promoted secondary metabolite accumulation to combat oxidative stress.

### 4.4 Transcriptomic analysis links AMF to osmoprotectant and antioxidant pathways

At the transcriptomic level, several genes highly upregulated in AMF-inoculated plants were directly linked to osmotic adjustment and oxidative stress tolerance (**Figures 5A, B**). Among them, *CMO* (choline monooxygenase) regulates glycine betaine biosynthesis, a crucial osmoprotectant under salinity (Russell et al., 1998). Similarly, aspartic proteinase inhibitor genes encode protease inhibitors that prevent excessive protein degradation, protecting photosynthetic proteins and minimizing oxidative damage (Barbole et al., 2022). Another upregulated gene, *CBSCBS2*, is implicated in signal transduction cascades regulating gene expression and carbohydrate metabolism (STRING, 2025), consistent with a potential role of AMF in modulating metabolic homeostasis under stress. In contrast, stress-related genes such as *C2H2-ZFP*, which act as central regulators of transcriptional, hormonal, and ROS signaling pathways (Liu et al., 2022), were strongly downregulated in AMF-inoculated plants, suggesting an attenuation of salinity-induced stress responses. Similarly, the ABA receptor gene *PYL4*, which mediates abscisic acid-dependent stress signaling, was significantly downregulated, indicating a shift toward enhanced metabolic stability rather than stress-induced signaling activation (Karimi et al., 2021). KEGG pathway enrichment analysis further reinforced the metabolic findings, with oxidative phosphorylation, carbon metabolism, and the TCA among the most enriched pathways in AMF-inoculated plants (**Figures 5C, D**). These pathways play a central role in the biosynthesis of key osmoprotectants, providing essential precursors for stress adaptation.

### 4.5 WGCNA identifies gene networks associated with AMF-mediated stress adaptation

WGCNA analysis provided insights into transcriptional patterns correlated with metabolites and traits involved in osmoprotectant and antioxidant pathways under salinity stress. The darkslateblue module, which exhibited higher expression in AMF-inoculated plants, was positively correlated with several metabolites, including PEP, 3PGA, 2-oxoglutarate, malate, oxalic acid, ADP, alanine, glutamate, isoleucine, aspartate, ferulic acid, and benzoic acid (**Figure 6**). Several genes within this module are known to be involved in the biosynthesis of these key metabolites, and their co-expression together with the positive module-metabolite correlations suggest coordinated transcriptional and metabolic responses associated with AMF inoculation. Among them, *PHS2* is known to be involved in starch breakdown into glucose-1-phosphate, providing substrates for PEP biosynthesis (Ma et al., 2013). *PPD*, an ATP-dependent pyruvate-to-PEP converter, plays a role in glycolysis and gluconeogenesis, contributing to energy balance under stress (Chastain et al., 2002). *TD1* catalyzes the conversion of threonine to 2-oxobutanoate, a precursor in isoleucine biosynthesis (Yeo et al., 2023). Furthermore, *MASY*_*GOSHI*, encoding malate synthase (*MS*), is involved in glyoxylate and oxalic acid metabolism and may contribute to osmotic balance and ROS scavenging (**Table 3**) (Cornah and Smith, 2002).

In contrast, genes clustered in the darkorange2 module, which showed lower expression in AMF-inoculated plants, were negatively correlated with glucose-6-P, fructose-6-P, hexose-P, PEP, 3PGA, ADP, aspartate, glutamate, alanine, isoleucine, malate, 2-oxoglutarate, oxalic acid, ferulic acid, and benzoic acid, suggesting that this module may be associated with pathways related to the turnover or degradation of these metabolites. The reduced expression of this module in AMF-inoculated plants may therefore be consistent with the maintenance of higher metabolite levels. Supporting this interpretation, several genes in darkorange2 encode enzymes involved in catabolic processes: *MAON*_*SOLTU* encodes an NAD-dependent malic enzyme (*ME*) that catalyzes the conversion of malate to pyruvate (Jenner et al., 2001). *At4g26910* encodes the E2 subunit of the 2-oxoglutarate dehydrogenase complex (2-OGDH), which is involved in 2-oxoglutarate turnover in the TCA cycle (Condori-Apfata et al., 2021). Additionally, *At5g26710* facilitates glutamate utilization for protein biosynthesis (Brandão et al., 2009), while *BCE2* and *At5g09300*, both involved in isoleucine degradation, are associated with amino acid metabolism processes related to stress responses (Mooney et al., 2000; Peng et al., 2015). Notably, several of the enriched or preserved metabolites, such as glutamate, 2-oxoglutarate, malate, and PEP, are direct or indirect precursors for the proline biosynthesis pathway. Their coordinated accumulation, together with module-level expression patterns, suggests a potential redirection of carbon and nitrogen flux toward proline biosynthesis under salinity, which may contribute to the increased proline levels observed in AMF-inoculated plants.

## 5 Conclusion

**Figure 7:**
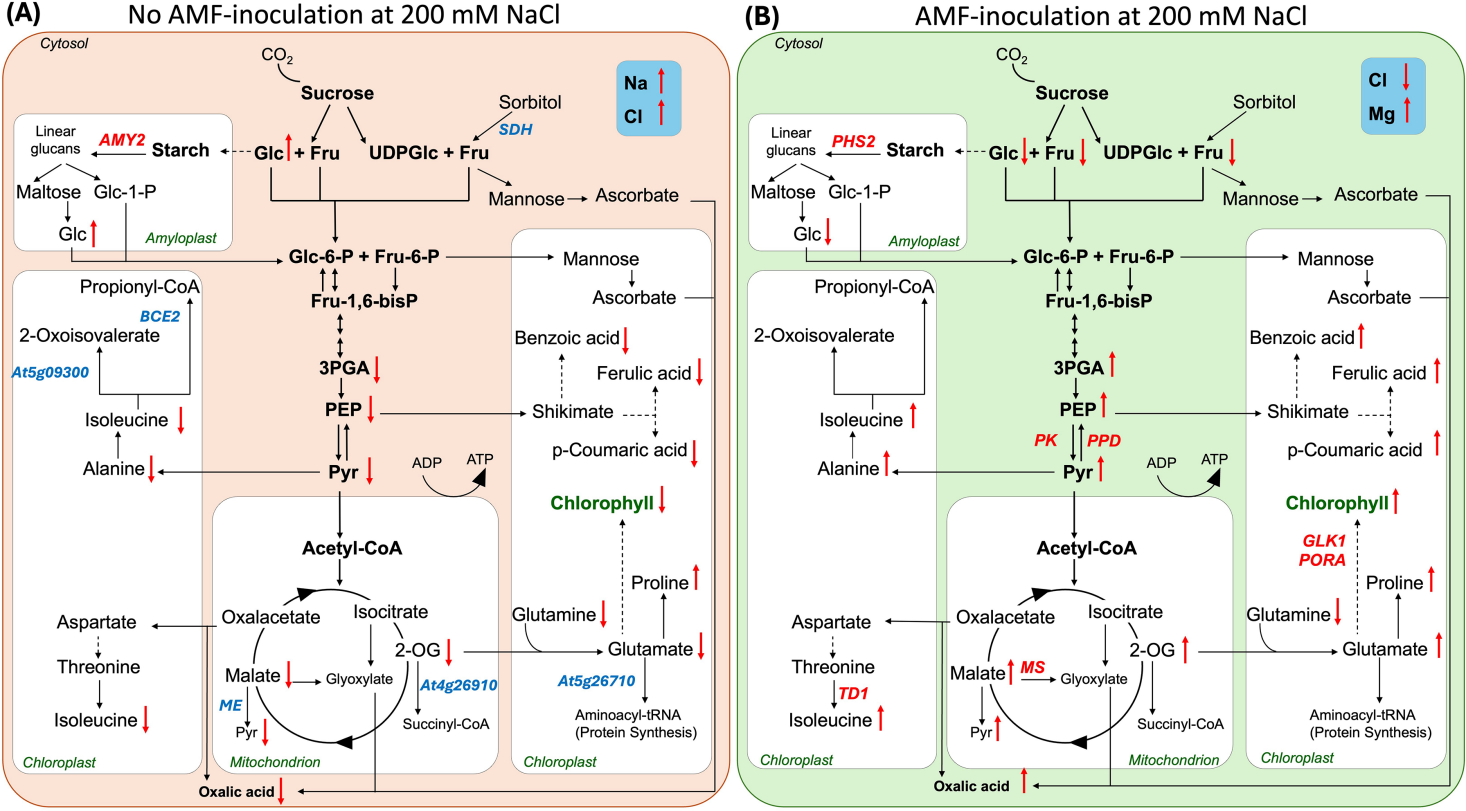
Summary of metabolic pathway alterations in quinoa under salinity stress (200 mM NaCl) and AMF inoculation (50 g AMF inoculum). **(A)** salinity-induced metabolic and nutrient changes in non-inoculated plants. **(B)** AMF-driven metabolic and nutrient adjustments under salinity. Metabolites and nutrients are labeled in black, with gene names in red representing higher expression in AMF plants, while those in blue indicate higher expression in non-inoculated plants. Solid lines indicate direct biosynthesis, while dashed lines represent intermediate steps. Arrows in red indicate increases or decreases in metabolite or nutrient concentrations. Glc, glucose; Fru, fructose; UDPGlc, uridine diphosphate glucose; 3PGA, 3-phosphoglycerate; PEP, phosphoenolpyruvate; Pyr, pyruvate.

This study provides an integrative multi-omics perspective on the role of AMF in quinoa’s early vegetative responses to salinity. AMF potentially contributed to improved stress adaptation by reducing Cl^-^ accumulation, maintaining Chl levels, and modulating central carbon metabolism. AMF inoculation promoted sustained accumulation of key metabolic intermediates, amino acids, and phenolic compounds, along with upregulation of genes and pathways involved in carbon metabolism, photosynthesis, and osmoprotection (**Figure 7**). While these findings highlight coordinated metabolic and transcriptional adjustments, additional physiological measurements are needed to better link these responses to whole-plant performance. Furthermore, the mechanisms underlying the AMF-mediated reduction in Cl^-^ accumulation remain to be elucidated, as Cl^-^ dynamics in roots, subcellular compartmentalization, and the involvement of specific ion transporters were not assessed.

Future studies integrating physiological, metabolic, ionic, and transcriptomic approaches, together with field validation, will be essential to further elucidate these mechanisms and evaluate the agronomic relevance of AMF-mediated salinity resilience in quinoa under realistic conditions.

## Data Availability

The datasets presented in this study can be found in online repositories. The names of the repository/repositories and accession number(s) can be found below: https://www.ebi.ac.uk/ena, PRJEB104472.
