# Supplementary material for: Mycorrhizal inoculation modulates metabolism and gene networks to enhance salinity tolerance in quinoa
Source: Front Plant Sci. 2026 May 20;17:1831758. doi: 10.3389/fpls.2026.1831758 (PMC13229980; doi:10.3389/fpls.2026.1831758)
Supplement: Supplementary file 2 [file DataSheet1.pdf]

Supplementary Material

1. Supplementary Figures

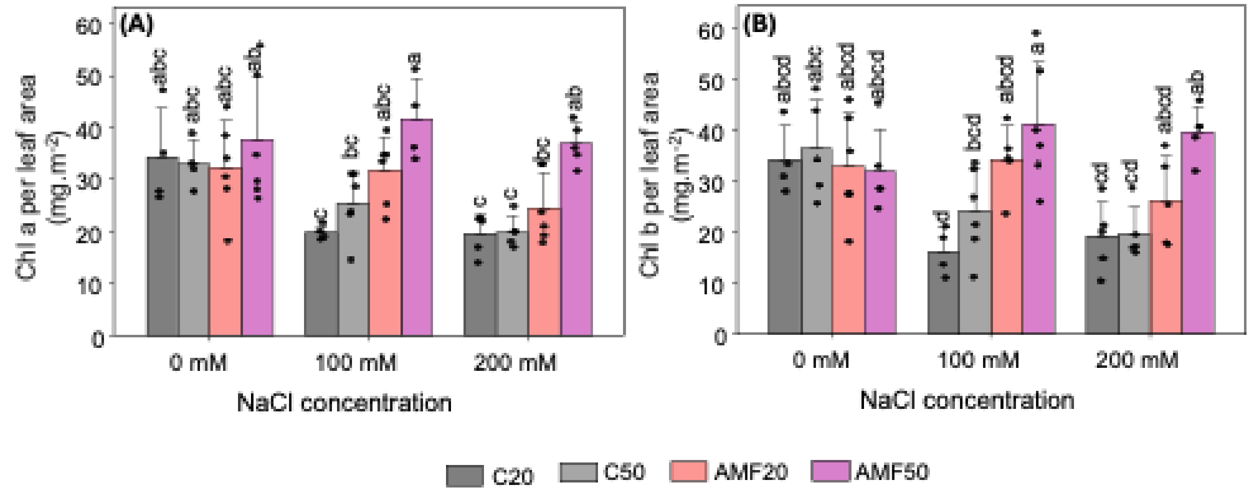

Supplementary Figure 1 Changes in Chl a and Chl b concentrations in quinoa leaves under different arbuscular mycorrhizal fungi (AMF) treatments (C20 and C50: 20 g and 50 g sterilized inoculum; AMF20 and AMF50: 20 g and 50 g AMF inoculum) and salinity levels (0. 100. and 200 mM NaCl). (A) Chl a. (B) Chl b. Data are presented as means + SD, n = 3-6. Dots represent individual biological replicates. Different lowercase letters indicate significant differences among all combinations of treatment and salinity ($p < 0.05$), based on Tukey's HSD test. Chl: chlorophyll.

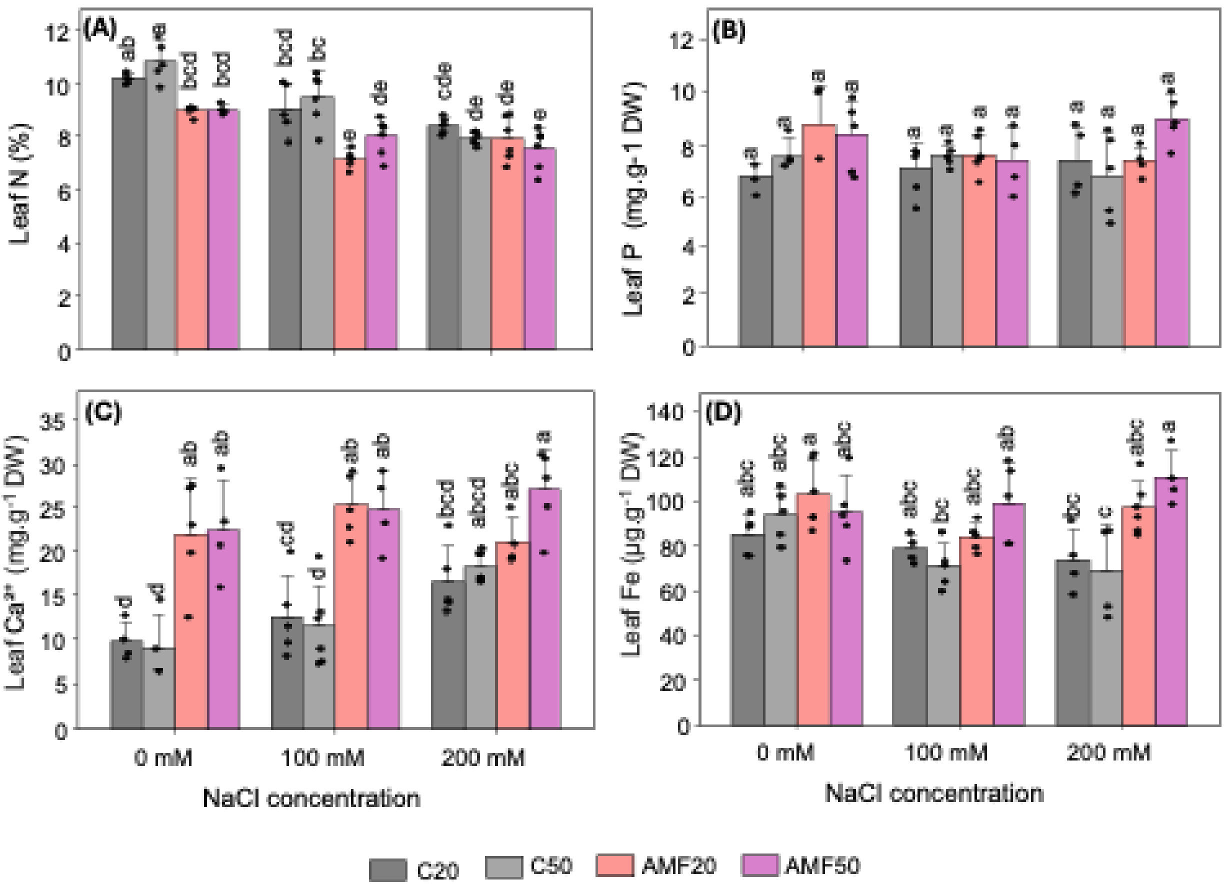

Supplementary Figure 2 Macro- and micronutrient concentrations in quinoa leaves subjected to different arbuscular mycorrhizal fungi (AMF) treatments (C20 and C50: 20 g and 50 g sterilized inoculum; AMF20 and AMF50: 20 g and 50 g AMF inoculum) and salinity levels (0. 100. and 200 mM NaCl). (A) Nitrogen. (B) Phosphorus. (C) Calcium. Data are presented as means + SD, n = 3-6. Dots represent individual biological replicates. Different lowercase letters indicate significant differences among all combinations of treatment and salinity ($p < 0.05$), based on Dunn's test. DW: dry weight.

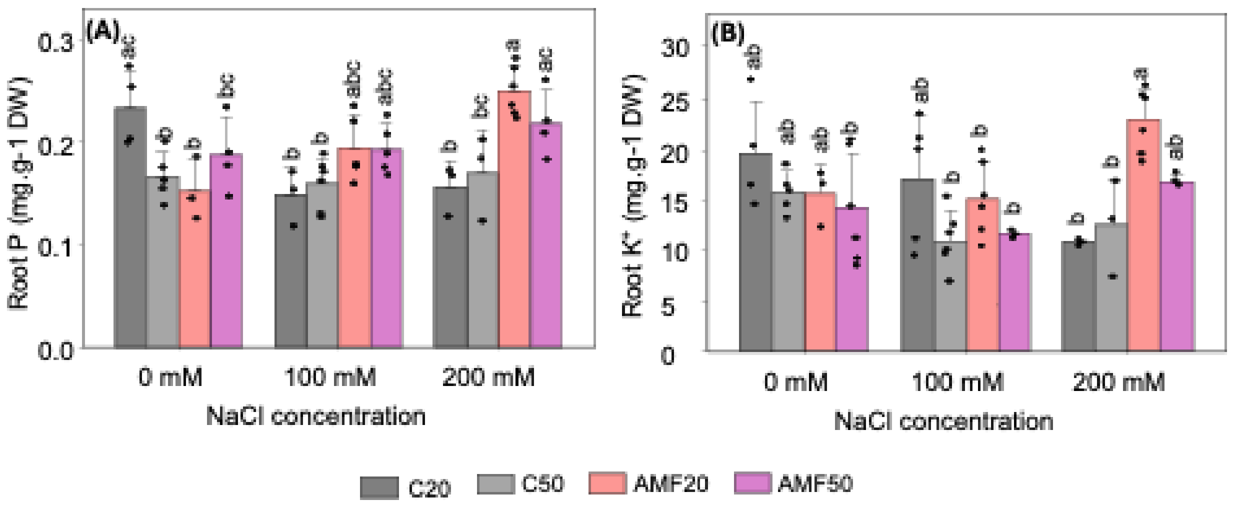

Supplementary Figure 3 Macronutrient concentrations in quinoa leaves subjected to different arbuscular mycorrhizal fungi (AMF) treatments (C20 and C50: 20 g and 50 g sterilized inoculum; AMF20 and AMF50: 20 g and 50 g AMF inoculum) and salinity levels (0. 100. and 200 mM NaCl). (A) Phosphorus. (B) Potassium. Data are presented as means + SD, n = 3-6. Dots represent individual biological replicates. Different lowercase letters indicate significant differences among all combinations of treatment and salinity ($p < 0.05$), based on Dunn's test. DW: dry weight.

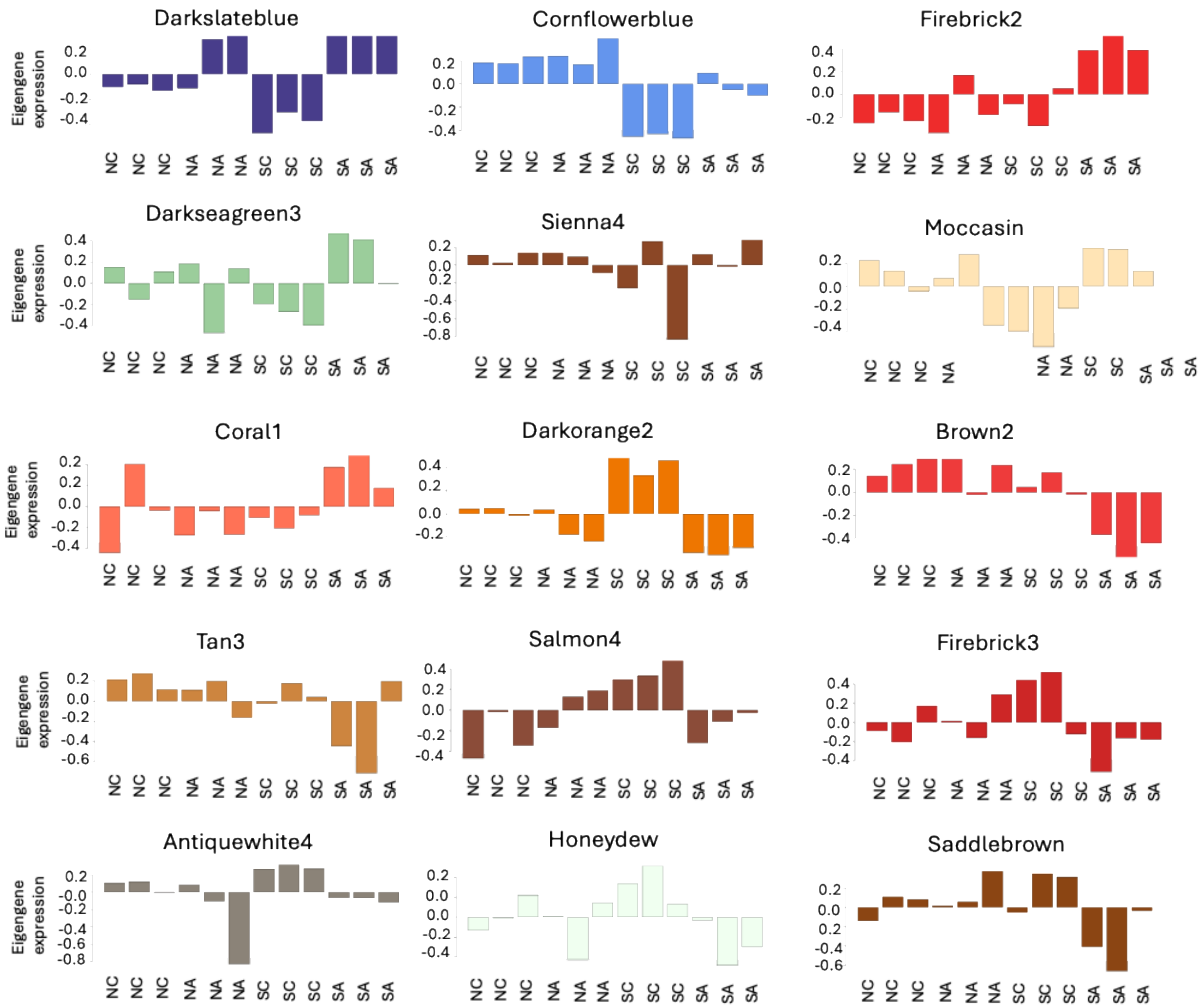

Supplementary Figure 4 Eigengene expression patterns of WGCNA-derived modules in quinoa under salinity stress (200 mM) and AMF treatment (AMF50: 50 g AMF inoculum). Eigengene modules were identified using a soft-thresholding power of 31. clustering strongly co-expressing genes into distinct modules. A total of 23.016 genes from the RNA sequencing (12 samples) were analyzed using WGCNA. N: no salinity. S: salinity. A: AMF-inoculated. C: non-inoculated control.

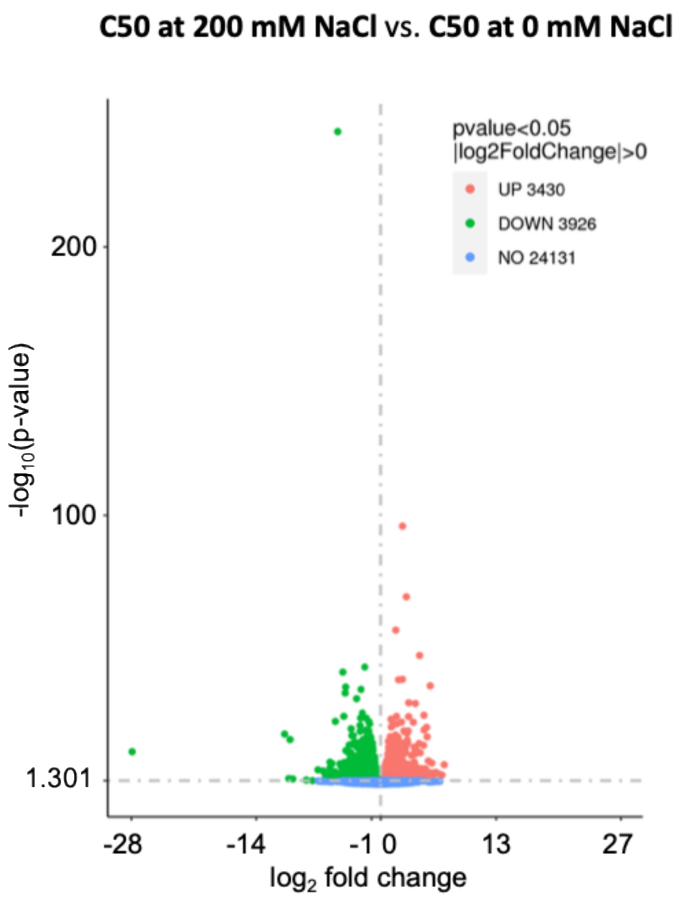

Supplementary Figure 5 Volcano plots displaying differentially expressed genes between control quinoa plants under no salinity vs salinity stress conditions. categorized as upregulated or downregulated ($p < 0.05$. $|\log_2$ fold change$| > 1$).

2. Supplementary Tables

Supplementary Table 1 Growth responses of quinoa to different salinity levels (0, 100, and 200 mM NaCl) and low AMF inoculation (10 g) (previous experiment). Data are presented as means ± SD, n = 6-8 biological replicates. Different lowercase letters in the same column indicate significant differences ($p < 0.05$), based on Tukey's HSD test (dry wight), and Dunn's test (plant height).

Parameter	Treatment	0 mM	200 mM	400 mM
**Plant height (cm)**	Control	73.71 ± 9.20 c<br>57.29 ± 7.97 bc	47.63 ± 9.46 ab<br>51.75 ± 8.36 ab	46.00 ± 4.38 a<br>48.25 ± 7.03 ab
**Dry weight (g)**	AMF	26.33 ± 3.01 a<br>22.43 ± 2.51 b	23.63 ± 2.20 ab<br>24.88 ± 1.89 ab	16.71 ± 1.50 c<br>16.25 ± 1.83 c

Supplementary Table 3 Amino acid concentrations in quinoa leaves subjected to different arbuscular mycorrhizal fungi (AMF) treatments (C20 and C50: 20 g and 50 g sterilized inoculum; AMF20 and AMF50: 20 g and 50 g AMF inoculum) and salinity levels (0, 100, and 200 mM NaCl). Data are presented as means ± SD, n = 3-6 biological replicates. Different lowercase letters indicate significant differences among all combinations of treatment and salinity ($p < 0.05$), based on Dunn's test.

Amino acid (nmol.g^{-1} FW)	Treatment	0 mM	100 mM	200 mM
**GABA**	C20	31 ± 8 b	117 ± 88 cd	199 ± 96 d
	C50	48 ± 17 ab	67 ± 24 abc	185 ± 101 cd
	AMF20	69 ± 30 abcd	74 ± 27 acd	55 ± 27 ab
	AMF50	45 ± 15 ab	73 ± 32 abc	67 ± 15 ab
**Glutamine**	C20	14461 ± 1215 abc	7827 ± 4971 bcde	9308 ± 897.4 cde
	C50	13664 ± 1323 abc	10363 ± 2501 bcd	8003 ± 2520 def
	AMF20	15686 ± 3023 a	2838 ± 752 g	5962 ± 3207 efg
	AMF50	14635 ± 1530 ab	4984 ± 2295 afg	4173 ± 2270 fg
**Histidine**	C20	1384 ± 465 abcd	1576 ± 1066 ab	1835 ± 477 ab
	C50	1369 ± 740 abc	2450 ± 711 a	1574 ± 1110 ab
	AMF20	284 ± 196 cd	174 ± 131 d	781 ± 569 bcd
	AMF50	406 ± 355 cd	258 ± 237 d	453 ± 188 cd

Supplementary Table 4. Gene distribution across co-expression modules identified by WGCNA in quinoa under arbuscular mycorrhizal fungi (AMF) treatment and salinity stress. A total of 42 co-expression modules were identified using a soft-thresholding power of 31. based on. 23.016 genes were obtained from RNA sequencing of 12 samples. Modules are categorized by their assigned color and gene count.

Module number	Module color	Gene count
1	antiquewhite2.csv	106
2	antiquewhite4.csv	413
3	blue3.csv	474
4	blue4.csv	275
5	brown1.csv	72
6	brown2.csv	2319
7	chocolate3.csv	3283
8	chocolate4.csv	84
9	coral.csv	106
10	coral1.csv	141
11	coral3.csv	104
12	coral4.csv	452
13	cornflowerblue.csv	1921
14	darkolivegreen1.csv	1759
15	darkolivegreen4.csv	116
16	darkorange2.csv	1103
17	darkseagreen3.csv	483
18	darkseagreen4.csv	146
19	darkslateblue.csv	1546
20	deeppink.csv	1006
21	firebrick2.csv	1826

Number	Module color	Gene count
22	firebrick3.csv	509
23	green4.csv	752
24	grey.csv	245
25	honeydew.csv	145
26	honeydew1.csv	403
27	lavenderblush1.csv	164
28	lavenderblush2.csv	298
29	lightpink1.csv	65
30	lightsteelblue.csv	237
31	diumorchid.csv	608
32	diumpurple2.csv	122
33	moccasin.csv	296
34	palevioletred2.csv	113
35	saddlebrown.csv	219
36	salmon4.csv	155
37	sienna4.csv	102
38	skyblue1.csv	320
39	slateblue1.csv	52
40	tan3.csv	139
41	violet.csv	209
42	yellow4.csv	128
	**TOTAL**	**23.016**
